# Supplementary material for: 9-Cyanopyronin probe palette for super-multiplexed vibrational imaging
Source: Nat Commun. 2021 Jul 26;12:4518. doi: 10.1038/s41467-021-24855-6 (PMC8313527; doi:10.1038/s41467-021-24855-6)
Supplement: Supplementary file 1 — Supplementary Information [file 41467_2021_24855_MOESM1_ESM.pdf]

Supplementary Information

9-Cyanopyronin Probe Palette for Super-Multiplexed Vibrational Imaging

Yupeng Miao[1], Naixin Qian[1], Lixue Shi[1], Fanghao Hu[1], and Wei Min[*,1,2]

[1]Department of Chemistry, Columbia University, New York, NY 10027, USA

[2]Kavli Institute for Brain Science, Columbia University, New York, NY 10027, USA

*Email: wm2256@columbia.edu

Table of contents

Supplementary Figures and Tables **2**
Supplementary Figure1 2
Supplementary Table 1 3
Supplementary Figure 2 4
Supplementary Figure 3 5
Supplementary Figure 4 6
Supplementary Figure 5 7
Supplementary Figure 6 8
Supplementary Table 2 9
Supplementary Figure 7 10
Supplementary Figure 8 11
Supplementary Table 3 12
Supplementary Table 4 13
Synthesis notes **14**
General experimental methods 14
Preparation of related precursors 15
Synthesis of O-cored MARS model compounds (**1-5**) 18
Synthesis of O-cored MARS NHS esters (**6a-d**, **9a-d**, **10a-d**) 23
Synthesis of C-cored MARS NHS esters (**7a-d**) 32
Synthesis of Si-cored MARS NHS esters (**8a-d**) 35
Synthesis of functionalized MARS probes for multiplexed imaging 35
Reference **44**

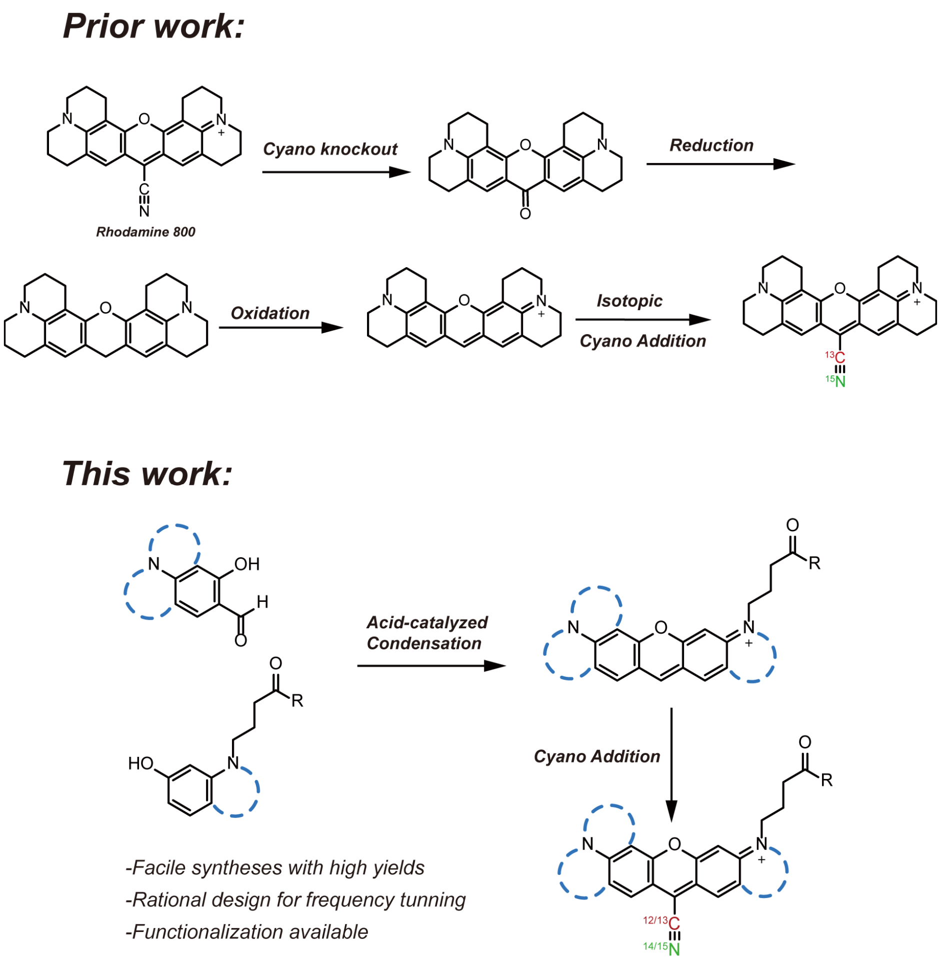

Supplementary Figure 1 Comparison between previously reported synthesis and current work on synthesis of isotope-doped Rhodamine 800.

Supplementary Table 1. Synthesis of O-cored 9-cyanopyronins

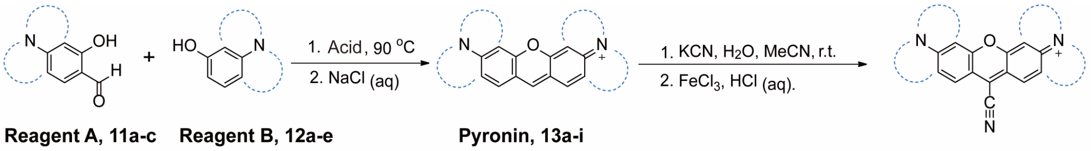

Reagent A, 11a-c **Reagent B, 12a-e** **Pyronin, 13a-i** **O-cored MARS Dye**

Reagent A		Reagent B		Acid	Pyronin Intermediate		Yield (%)	MARS dye		Yield (%)
**11a**	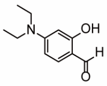	**12a**	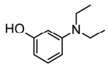	H_3PO_4	**13a**	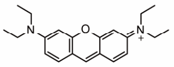	64	**1**	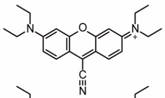	88
**11a**	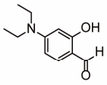	**12b**	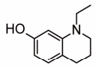	MsOH	**13b**	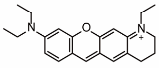	72	**2**	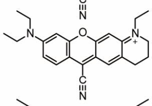	89
**11b**	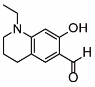	**12b**	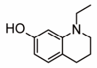	MsOH	**13c**	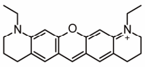	75	**3**	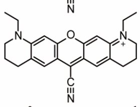	82
**11c**	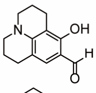	**12b**	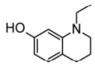	MsOH	**13d**	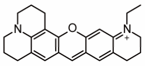	65	**4**	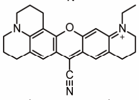	71
**11c**	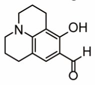	**12c**	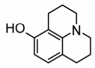	H_3PO_4	**13e**	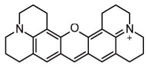	78	**5**	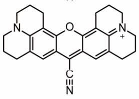	87
**11d**	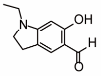	**12d**	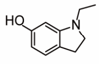	MsOH	**13f**	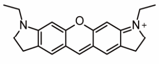	50	**14**	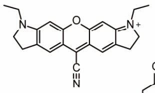	48
**11a**	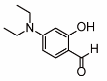	**12e**	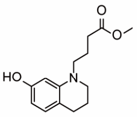	MsOH	**13g**	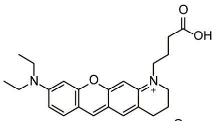	56	**15**	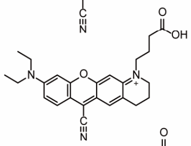	81
**11b**	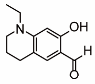	**12e**	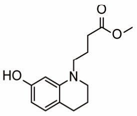	MsOH	**13h**	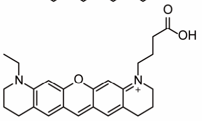	51	**16**	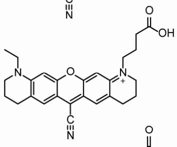	69
**11c**	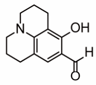	**12e**	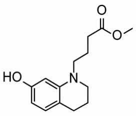	MsOH	**13i**	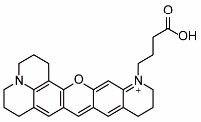	66	**17**	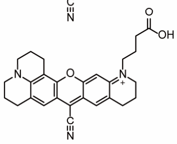	77

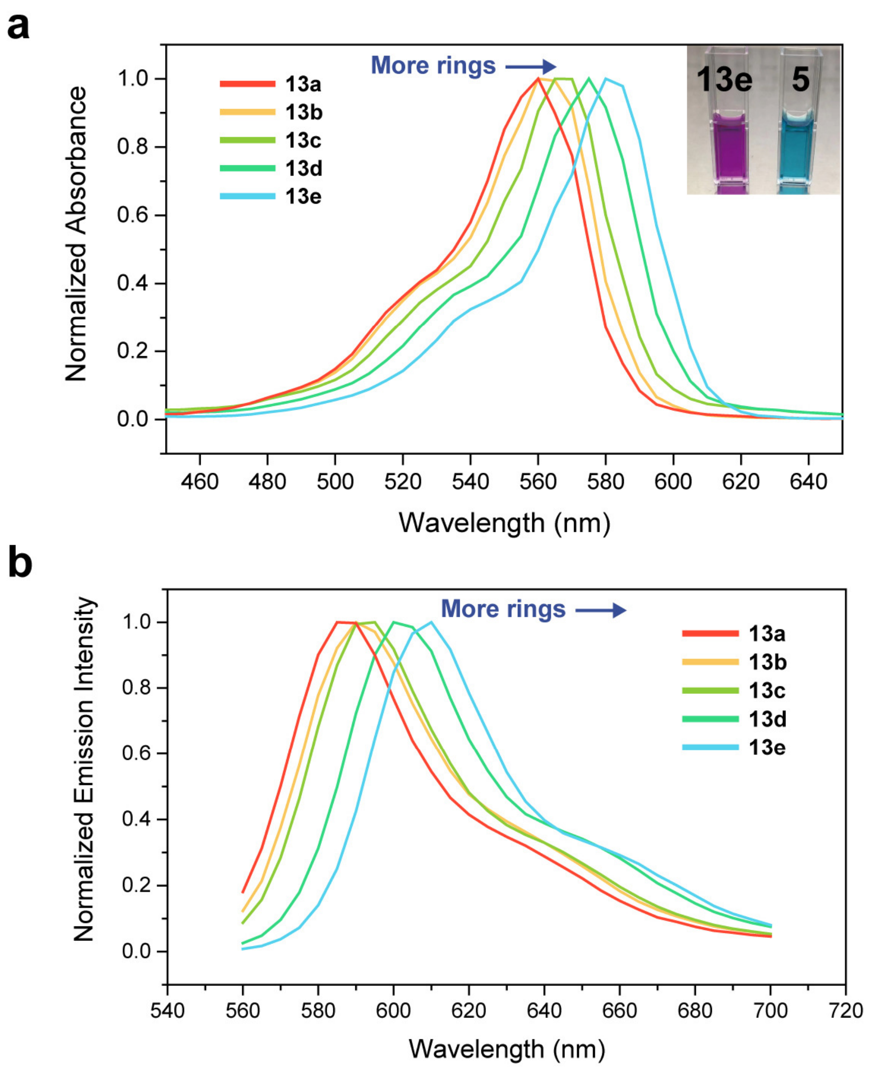

Supplementary Figure 2 Normalized absorbance and fluorescence emission spectra of five O-cored pyronin intermediates. (a) Normalized absorption spectra taken in DMSO. Inset: appearance of 100 μM molecule **13e** and **5** in PBS buffer (pH=7.4) (b) Normalized fluorescence emission spectra measured in DMSO. Excitation at 520 nm.

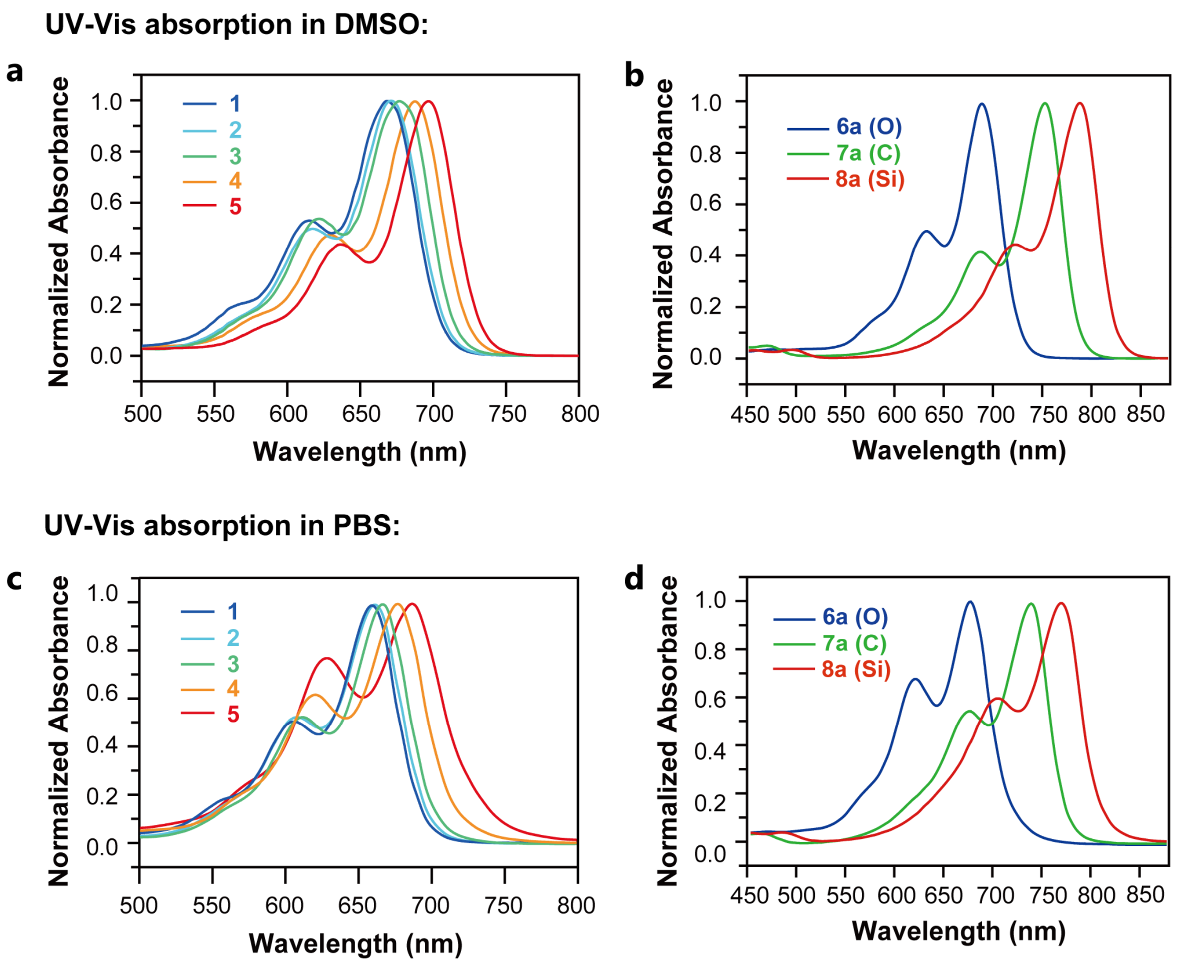

Supplementary Figure 3 (a) Normalized absorption spectra of **1-5** measured in DMSO showing the gradual shift in maximum wavelength. (b) Normalized absorption spectra of O-, C-, and Si- cored MARS dyes. (c) and (d): corresponding absorption spectra measured in PBS buffer (10 µM).

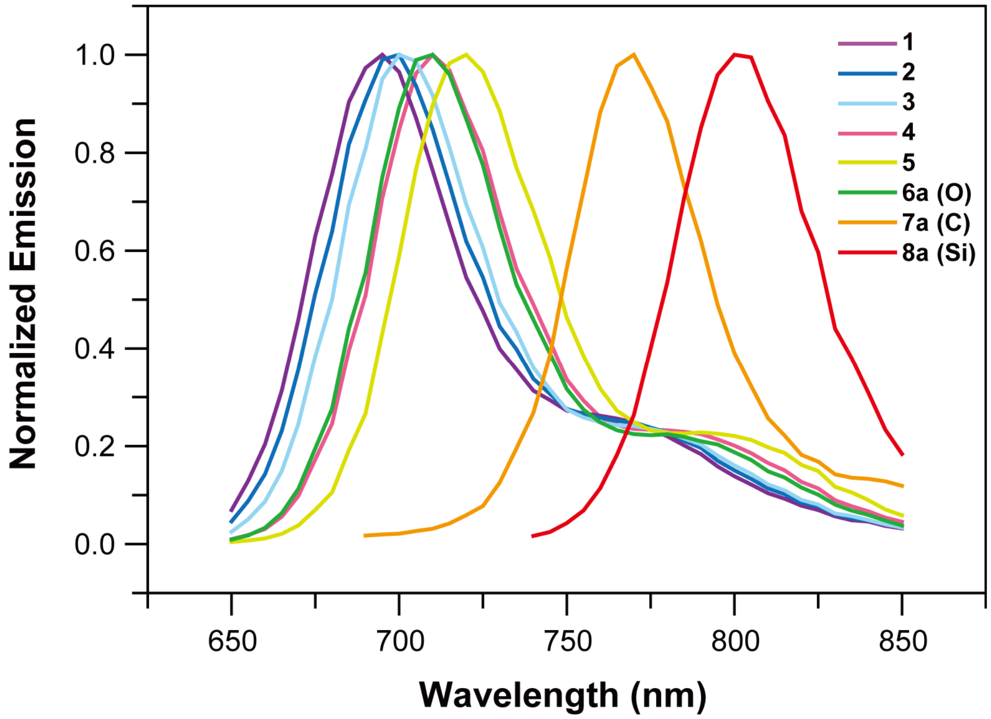

Supplementary Figure 4. Normalized fluorescence emission spectra of representative 9-cyanopyronin dyes in DMSO.

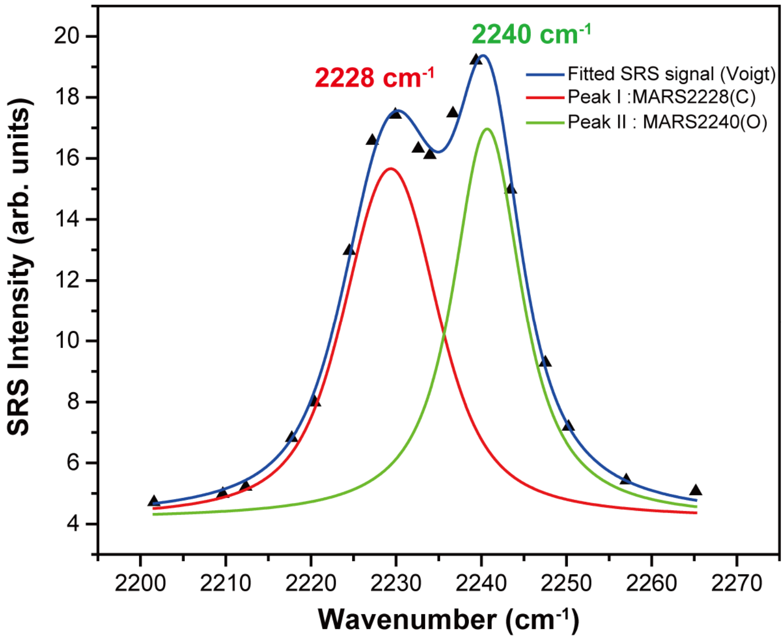

Supplementary Figure 5. In-vitro unmixing of **MARS2228-NHS** and **MARS2240-NHS**. Two probes were diluted with DMSO to obtain a mixture displaying similar SRS intensities. The deconvolution was performed based on Voigt multi-peak fitting. Two separated peaks show exact the same Raman shifts as two probe components.

a

Dye	Core atom	Nitrile	# of rings	Sidechain groups	λ_{abs} (nm)	λ_{em} (nm)	Raman shifts (cm^{-1})
**1**	O	C≡N	0	-Et	670	701	2241
**2**			1		675	704	2240
**3**			2		680	710	2239
**4**			3		690	722	2238
**5**			4	-	700	734	2237
**9a**	O	C≡N	1		675	705	2240
**9b**		$C\equiv^{15}N$	1		675	705	2212
**9c**		$^{13}C\equiv N$	1		675	705	2186
**9d**		$^{13}C\equiv^{15}N$	1		675	705	2157
**10a**	O	C≡N	2		680	710	2239
**10b**		$C\equiv^{15}N$	2		680	710	2211
**10c**		$^{13}C\equiv N$	2		680	710	2185
**10d**		$^{13}C\equiv^{15}N$	2		680	710	2156
**6a**	O	C≡N	3		690	720	2238
**6b**		$C\equiv^{15}N$	3		690	720	2210
**6c**		$^{13}C\equiv N$	3		690	720	2184
**6d**		$^{13}C\equiv^{15}N$	3		690	720	2155
**7a**	C	C≡N	3		760	785	2228
**7b**		$C\equiv^{15}N$	3		760	785	2200
**7c**		$^{13}C\equiv N$	3		760	785	2176

Dye	Core atom	Nitrile	# of rings	Sidechain groups	λ_{abs} (nm)	λ_{em} (nm)	Raman shifts (cm^{-1})
**7d**		$^{13}C\equiv^{15}N$	3		760	785	2147
**8a**	Si	C≡N	3		790	810	2222
**8b**		$C\equiv^{15}N$	3		790	810	2197
**8c**		$^{13}C\equiv N$	3		790	810	2172
**8d**		$^{13}C\equiv^{15}N$	3		790	810	2139
**MARS2242**	O	C≡N	0	-Me	667	700	2242
**MARS2214**		$C\equiv^{15}N$	0		667	700	2214
**MARS2186**		$^{13}C\equiv N$	0		667	700	2186
**MARS2159**		$^{13}C\equiv^{15}N$	0		667	700	2159
**MARS2209**	O	$C\equiv^{15}N$	4	-	700	734	2209
**MARS2183**		$^{13}C\equiv N$	4		700	734	2183
**MARS2154**		$^{13}C\equiv^{15}N$	4		700	734	2154
**MARS2233**	C	C≡N	0	-Me	735	760	2233
**MARS2204**		$C\equiv^{15}N$	0		735	760	2204
**MARS2179**		$^{13}C\equiv N$	0		735	760	2179
**MARS2152**		$^{13}C\equiv^{15}N$	0		735	760	2152
**MARS2225**	Si	C≡N	0	-Me	760	781	2225
**MARS2199**		$C\equiv^{15}N$	0		760	781	2199
**MARS2173**		$^{13}C\equiv N$	0		760	781	2173
**MARS2145**		$^{13}C\equiv^{15}N$	0		760	781	2145

b

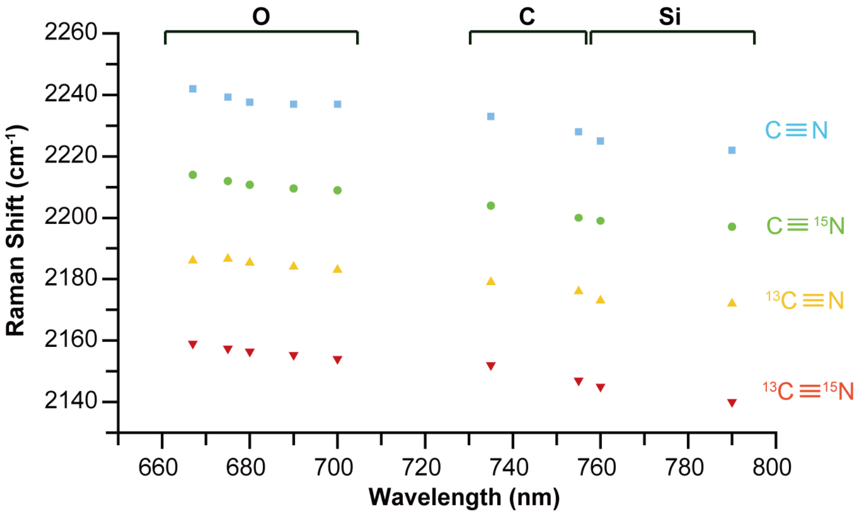

Supplementary Figure 6 (a) Comprehensive listing of spectroscopic properties of all MARS model compounds and NHS ester derivatives measured in DMSO. (b) Graphic presentation of MARS molecules' nitrile Raman shifts versus absorption maxima. Each spot represents a MARS compound.

Supplementary Table 2. Photophysical properties of newly synthesized MARS model compounds in PBS buffer.

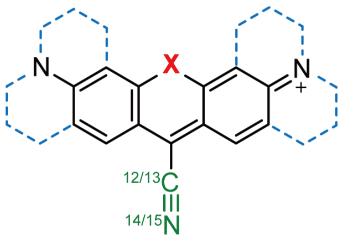

MARS	Nitrile	Core	Number of ring expansions	Measured Raman shift (cm^{-1})[a]	λ_{abs} (nm)[b]	RIE[c]
**1**		O	0	2247	655	60
**2**		O	1	2246	660	80
**3**	C≡N	O	2	2244	665	87
**4**		O	3	2242	675	90
**5**		O	4	2240	685	103
**6a (MARS2238-NHS)**	C≡N	O	3	2239	675	92
**MARS2239-NHS**	C≡N	O	2	2244	665	87
**MARS2240-NHS**	C≡N	O	1	2246	660	67
**7a (MARS2228-NHS)**	C≡N	C	3	2234	740	342
**8a (MARS2222-NHS)**	C≡N	Si	3	2225	770	683

a,b: Measured in PBS aqueous solution.
c: RIE: relative intensity v.s. EdU (5-ethynyl-2'-deoxyuridine) with same SRS acquisition parameters.

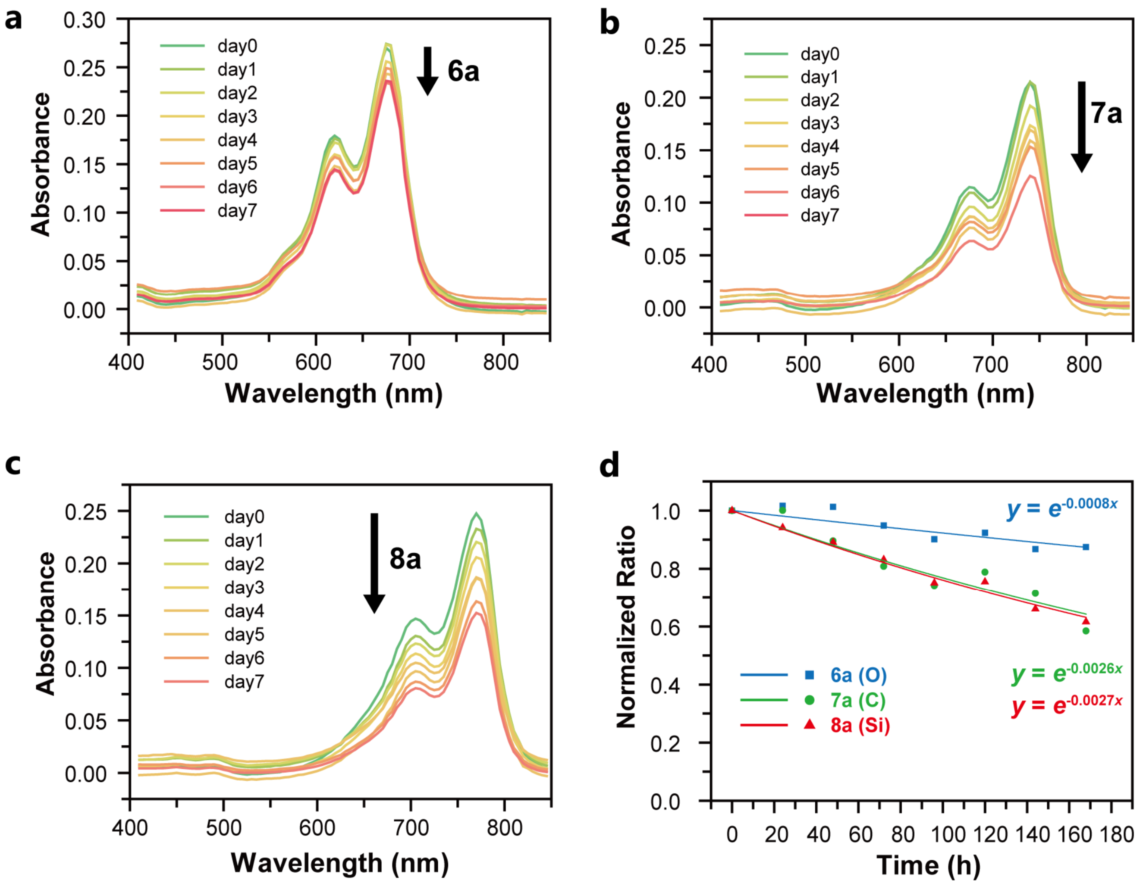

Supplementary Figure 7. Stability of 9-cyanopyronin probes in aqueous conditions. 10 μM of three representative 9-cyanopyronin probes in PBS buffer were stored in dark at room temperature and the absorbance was monitored every 24 h over one week.

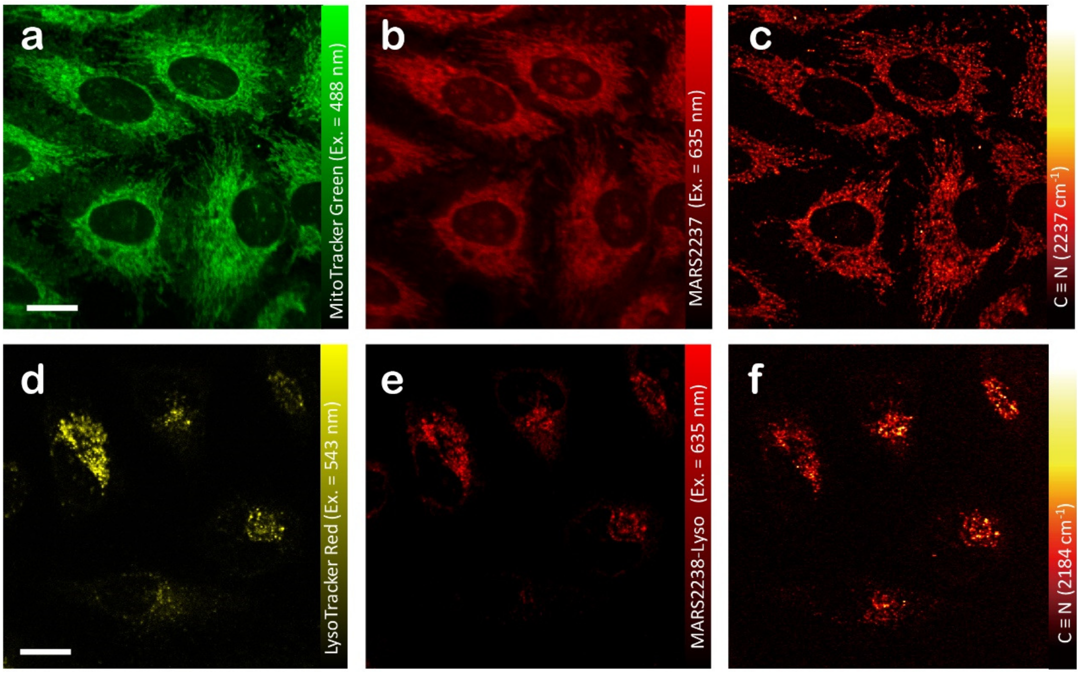

Supplementary Figure 8 Colocalization between commercial organelle markers and specialized MARS probes. (a) Mito-Tracker Green probes excited at 488 nm. (b) Fluorescence imaging of **MARS2237** excited at 635 nm. (c) SRS imaging of **MARS2237**. (c) Lyso-Tracker Red probes excited at 543 nm. (e) Fluorescence imaging of **MARS2184-Lyso**. (f) SRS imaging of **MARS2184-Lyso**. Scale bar: 20 μm.

Supplementary Table 3. Comparison between previously reported MARS molecules with newly developed molecules.

	Symmetry	Sidechain	Functionality	Examples
**Previously reported**	Symmetric	No	No	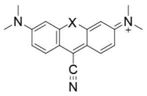 X= O, CMe_2, $SiMe_2$
**Previously reported but with improved chemistry**	Symmetric	No	No	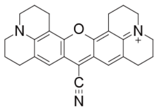
	Asymmetric	Yes	NHS-ester	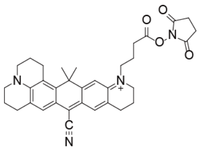
**Newly developed probes**	Asymmetric	No	No	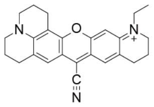
	Asymmetric	Yes	NHS-ester	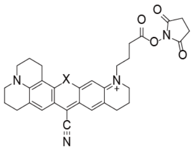 X= O, $SiMe_2$
	Asymmetric	Yes	Click chemistry	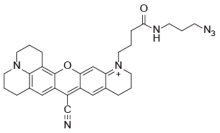
	Asymmetric	Yes	Organelle targeting	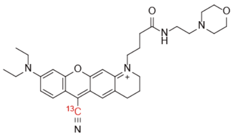
	Asymmetric	Yes	Cell skeleton targeting	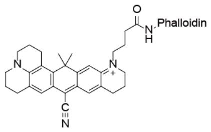
	Asymmetric	Yes	Lipid structure targeting	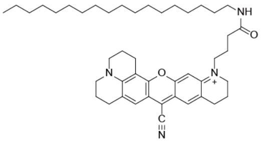

Supplementary Table 4. List of secondary antibodies used for conjugation.

Target	Vendor	Catalog#
Donkey anti-Mouse IgG (H+L)	Invitrogen	A16013
Donkey anti-Rat IgG (H+L)	Invitrogen	A18747
Donkey anti-Rabbit IgG (H+L)	Invitrogen	A31238
Donkey anti-Chicken IgY (H+L)	Invitrogen	SA1-72002
Donkey anti-Goat IgG (H+L)	Invitrogen	A16007
Donkey anti-Guinea Pig IgG (H+L)	Sigma-Aldrich	SAB3700384
Goat anti-Mouse IgG (H+L)	Invitrogen	31160
Goat anti-Rabbit IgG (H+L)	Invitrogen	31212

Synthesis notes

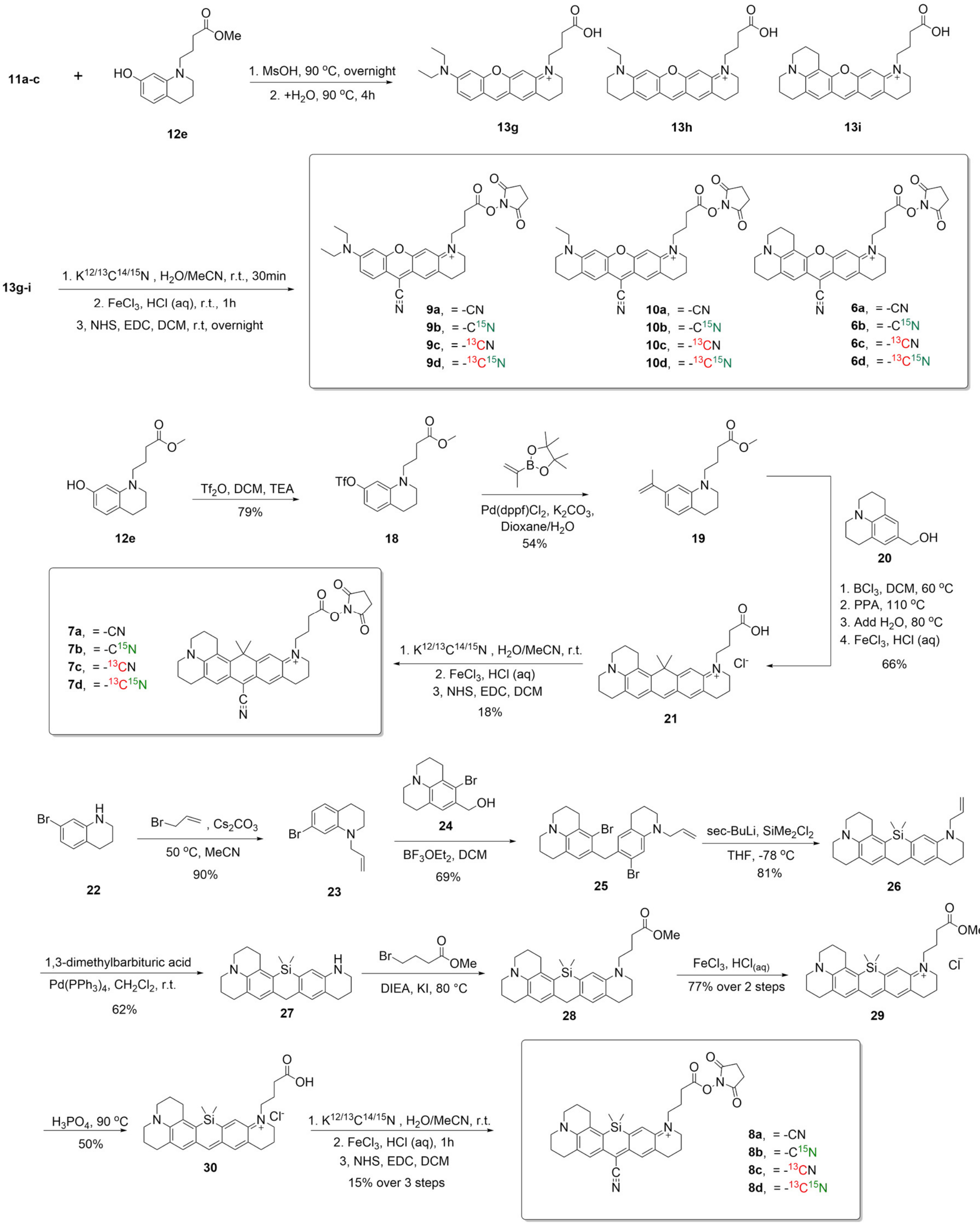

General experimental methods. Unless otherwise noted, all chemical reagents and solvents were purchased from Sigma-Aldrich, Fisher Scientific and Alfa-Aesar without further purification. Deuterated solvents and isotopic potassium cyanides were obtained from Cambridge Isotope Laboratories ($K^{13}CN$: CLM-297-PK, $KC^{15}N$: NLM-111-PK and $K^{13}C^{15}N$: CNLM-1961-PK). All cyanide-contaminated glassware was quenched with potassium permanganate ($KMnO_4$) before washing and disposal. Thin layer chromatography (TLC) was performed with MilliporeSigma™

silica gel 60 F_{254} coated aluminum-backed TLC sheets and visualized by UV-light at 254 nm. Normal phase flash chromatography was performed on Biotage Selekt system equipped with dual-channel UV-Vis detector. Preparative reverse phase high performance liquid chromatography (prep-HPLC) was performed on customized Gilson GX271 liquid handling system with dual-channel UV-Vis detector. Unless otherwise noted, all prep-HPLC used acetonitrile/H_2O with 0.1% trifluoroacetic acid (TFA) gradient as eluents. Typical gradient was from 20% to 90% within 15 min with 20 mL/min flow rate.

Nuclear magnetic resonance (NMR) spectra were recorded on Bruker 400 (400 MHz) or Bruker 500 (500 MHz) Fourier Transform (FT) NMR spectrometers in Department of Chemistry, Columbia University. Chemical shifts were calibrated using either residual undeuterated solvents: $CDCl_3$ (7.16 ppm for ^{1}H, 77.16 ppm for ^{13}C), CD_3OD (3.31 ppm for ^{1}H, 49.00 ppm for ^{13}C), DMSO-d_6 (2.50 ppm for ^{1}H, 39.52 ppm for ^{13}C), CD_3CN (1.94 ppm for ^{1}H, 118.26 ppm for ^{13}C) or trimethyl silane (TMS, 0.00 ppm for ^{1}H). NMR multiplicities were marked with following abbreviations: s=singlet, d=doublet, t=triplet, q=quartet, p=quintet, m=multiplet, and br=broad. High resolution mass spectra (HRMS) were obtained from a XEVO G2-XS Waters mass spectrometer equipped with a QTOF detector with multiple inlet and ionization capabilities. UV-Vis absorption spectra and fluorescence emission spectra were all recorded on a TECAN infinite-200 using 96-well plates as container.

Supplementary Figure 9. Preparation of related precursors for O-cored pyronins and MARS model compounds. Reaction conditions: (a) $LiAlH_4$, THF, reflux, overnight. (b) $NaBH_4$, acetic acid, acetaldehyde, r.t., 4 h. (c) Methyl 4-bromobutyrate, DIPEA, DMF, 90 °C, overnight. (d) $POCl_3$, DMF, 90 °C, 5 h. (e) $NaBH_4$, acetic acid, r.t., 4h.

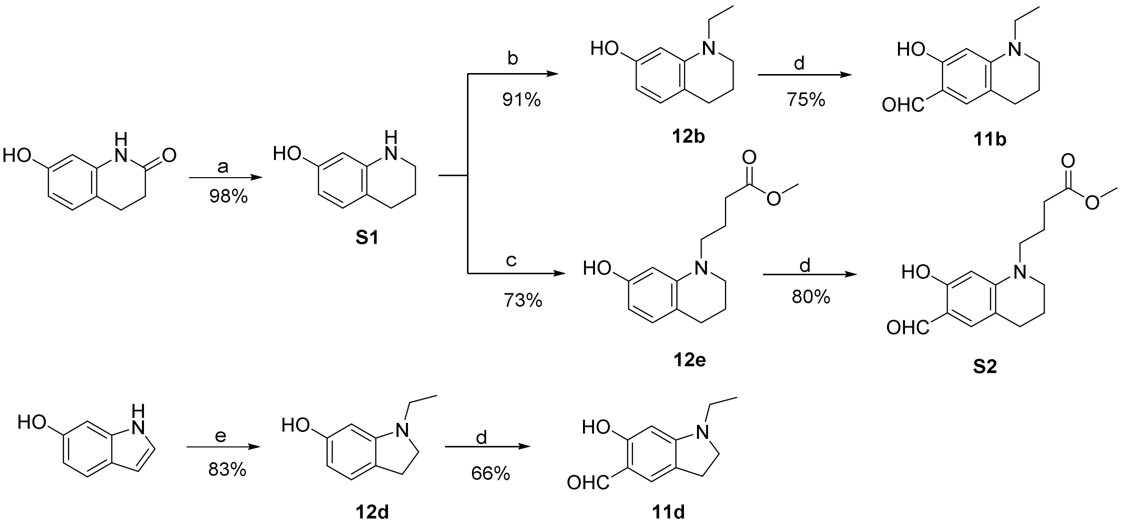

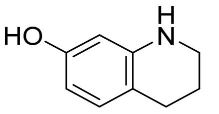

1,2,3,4-tetrahydroquinolin-7-ol (S1). S1 was synthesized according to previously published protocol.[1] A slurry of 7-hydroxy-3,4-dihydroquinolin-2(1H)-one (9.80 g, 60.0 mmol) in anhydrous THF (250 mL) was cooled within an ice bath and stirred vigorously. The slurry was carefully treated with $LiAlH_4$ (3.64 g, 96.9 mmol, 1.60 eq.) portionwise (bubbles evolved rapidly). Upon complete

addition, the reaction mixture was heated up and refluxed overnight. After cooled to room temperature, the reaction was cautiously quenched by addition of 100 mL saturated NH_4Cl solution. The resulted mixture was filtered through a pad of sand and washed with DCM (100 mL). The filtrate was further extracted with DCM (100 mL ×3) and the combined organic layers were dried over Na_2SO_4 and filtered. The solvent was then removed upon evaporation to afford **S1** as pale-yellow solid (8.80 g, 98%). The characterization data matches previous report. ^1H NMR (400 MHz, Chloroform-*d*) δ 6.80 (d, *J* = 8.1 Hz, 1H), 6.11 (dd, *J* = 8.1, 2.5 Hz, 1H), 5.98 (d, *J* = 2.5 Hz, 1H), 4.28 (br, 2H), 3.27 (t, *J* = 5.2 Hz, 2H), 2.69 (t, *J* = 6.4 Hz, 2H), 1.92 (m, 2H). HRMS (ASAP+) m/z Calcd. for $C_9H_{12}NO$ $[M+H]^+$: 150.0919. Found: 150.0915

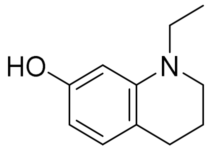

1-ethyl-1,2,3,4-tetrahydroquinolin-7-ol (12b). 12b was synthesized following previously published procedures.[1] A solution of **S1** (1.00 g, 6.70 mmol) in 25 mL acetic acid was slowly treated with $NaBH_4$ (1.02 g, 26.8 mmol, 4 eq.). After stirring at room temperature for 2 h, a solution of acetaldehyde (0.295 g, 6.70 mmol, 1 eq.) in 4 mL DCM was slowly injected into the reaction, which was closely monitored with TLC until starting material was fully consumed. The entire mixture was evaporated *in vacuo* to afford a thick residue followed by the addition of 60 mL saturated $NaHCO_3$. The pH of aqueous phase was adjusted to 7 with solid $NaHCO_3$ and extracted with ethyl acetate (30 mL ×3). The combined organic layers were dried over Na_2SO_4 and concentrated. The residue was purified with silica gel chromatography (Hexane → Hexane:EA = 2:1, v/v) to yield **12b** as white solid (1.08g, 91%). ^1H NMR (400 MHz, $CDCl_3$) δ 6.77 (d, *J* = 7.9 Hz, 1H), 6.12 (d, *J* = 2.4 Hz, 1H), 6.03 (dd, *J* = 7.9, 2.4 Hz, 1H), 4.47 (s, 1H), 3.30 (q, *J* = 7.1 Hz, 2H), 3.25 – 3.21 (m, 2H), 2.66 (t, *J* = 6.3 Hz, 2H), 1.97 – 1.87 (m, 2H), 1.13 (t, *J* = 7.1 Hz, 3H).

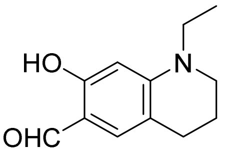

1-ethyl-7-hydroxy-1,2,3,4-tetrahydroquinoline-6-carbaldehyde (11b). To an Ar flushed flask was added 4 mL anhydrous N, N-dimethylformamide (DMF). After cooled to 0 °C, phosphoryl chloride ($POCl_3$, 0.33 mL, 3.6 mmol, 1.2 eq) was added to DMF and kept stirring for 30 min followed by the addition of **12b** (531 mg, 3.0 mmol) in 4 mL dry DMF. The temperature was raised to 90 °C and kept for 5 h while stirring. After cooled to room temperature, the reaction was quenched upon addition of 30 mL iced water and the pH was adjusted to 8 with solid $NaHCO_3$. The aqueous phase was extracted with ether (50 mL ×3) and the combined organic layers were dried over Na_2SO_4. The solvents were removed in vacuo to afford **11b** as brown solid, which has sufficient purity to be used in next step (461 mg, 75%). ^1H NMR (500 MHz, $CDCl_3$) δ 11.64 (s, 1H), 9.43 (s, 0H), 7.00 – 6.94 (m, 1H), 6.04 (s, 1H), 3.43 – 3.34 (m, 4H), 2.69 (td, *J* = 6.1, 1.0 Hz, 2H), 1.95 (dq, *J* = 7.4, 5.9 Hz, 2H), 1.21 (t, *J* = 7.1 Hz, 3H). ^{13}C NMR (126 MHz, $CDCl_3$) δ 191.5, 163.4, 151.9, 133.0, 114.8, 110.9, 95.5, 48.7, 46.0, 27.2, 21.8, 11.1. HRMS (ASAP+) m/z Calcd. for $C_{12}H_{16}NO_2$ $[M+H]^+$: 206.1181. Found: 206.1180

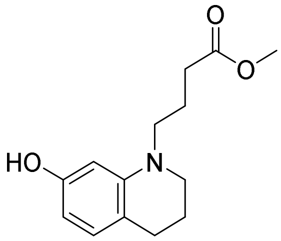

Methyl 4-(7-hydroxy-3,4-dihydroquinolin-1(2H)-yl)butanoate (12e). Compound **S1** (1.49 g, 10.0 mmol), methyl 4-bromobutyrate (2.17 g, 12.0 mmol, 1.2 eq.) and N,N-Diisopropylethylamine (DIPEA, 2.09 mL, 12.0 mmol, 1.2 eq) were dissolved in 20 mL anhydrous DMF. The solution was heated to 90 °C and stirred for 48 h. After cooled to r.t., the mixture was diluted with 50 mL saturated brine. The aqueous phase was extracted with DCM (50 mL ×3). The combined organic layers were dried upon Na_2SO_4 and concentrated to get crude product, which was further purified with chromatography to afford **12e** as yellowish oil (1.82 g, 73%). ^{1}H NMR (400 MHz, Chloroform-*d*) δ 6.79 (d, *J* = 8.0 Hz, 1H), 6.16 (d, *J* = 2.4 Hz, 1H), 6.08 (dd, *J* = 7.9, 2.4 Hz, 1H), 5.41 (s, 1H), 3.72 (s, 3H), 3.31 – 3.19 (m, 4H), 2.68 (t, *J* = 6.4 Hz, 2H), 2.39 (t, *J* = 7.2 Hz, 2H), 1.99 – 1.85 (m, 4H). ^{13}C NMR (126 MHz, $CDCl_3$) δ 174.1, 155.3, 145.8, 129.9, 115.0, 103.1, 98.4, 51.9, 51.1, 49.4, 31.4, 27.3, 22.3, 21.5. HRMS (ESI+) m/z Calcd. for $C_{14}H_{20}NO_3$ $[M+H]^+$: 250.1438. Found: 250.1442

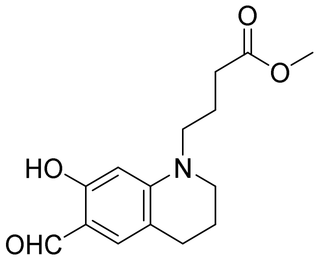

Methyl 4-(6-formyl-7-hydroxy-3,4-dihydroquinolin-1(2H)-yl)butanoate (S2). Similar to the synthesis of **1b**, $POCl_3$ (66 μL, 0.71 mmol, 1.2 eq.) was added to 1 mL anhydrous DMF cooled to 0 °C and stirred for 30 min, followed by the addition of **12e** (147 mg, 0.59 mmol) dissolved in 1 mL dry DMF. The mixture was then heated to 90 °C and kept stirring for 5 h. After cooled to r.t., the reaction was quenched with 20 mL saturated $NaHCO_3$ solution. The aqueous phase was extracted with DCM (20 mL ×3). The combined organic layers were dried over Na_2SO_4 and the solvents were removed thoroughly in vacuo. The residue was dissolved in 10 mL DCM and passed through a short pad of silica. The pad was washed with 100 mL DCM and 100 mL ethyl acetate. The collected fractions were evaporated to afford **S2** as yellowish oil (130 mg, 80%). ^{1}H NMR (500 MHz, $CDCl_3$) δ 9.45 (s, 1H), 6.98 (s, 1H), 6.05 (s, 1H), 3.72 (s, 3H), 3.41 – 3.35 (m, 4H), 2.72 – 2.67 (m, 2H), 2.40 (t, *J* = 7.2 Hz, 2H), 2.01 – 1.92 (m, 4H). ^{13}C NMR (126 MHz, $CDCl_3$) δ 191.8, 173.4, 163.5, 152.1, 133.3, 114.9, 111.3, 96.0, 77.4, 77.2, 76.9, 51.9, 50.9, 49.9, 31.1, 27.3, 21.9, 21.6.

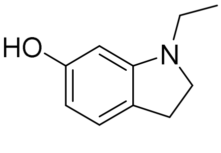

1-ethylindolin-6-ol (12d). To a solution of 6-hydroxyindole (1.0 g, 7.5 mmol) in 20 mL acetic acid, $NaBH_4$ (1.42 g, 37.3 mmol, 5 eq.) was added portionwise. The reaction was kept stirring for 4 h at room temperature until evaporated to obtain a thick brown residue. The crude was diluted with 50 mL saturated $NaHCO_3$ and further neutralized with solid $NaHCO_3$. The aqueous phase was extracted with ethyl acetate (50 mL ×3). The combined organic layers were washed with brine, dried over Na_2SO_4 and further purified by silica gel flash chromatography to afford **12d** as yellowish oil, which later crystalized slowly to pale yellow solid (1.02 g, 83%). ^{1}H NMR (500 MHz, $CDCl_3$) δ 6.88 (d, J = 7.8 Hz, 1H), 6.07 (dd, J = 7.8, 2.3 Hz, 1H), 5.99 (d, J = 2.2 Hz, 1H), 4.88 (br, 1H), 3.34 (t, J = 8.2 Hz, 2H), 3.09 (q, J = 7.2 Hz, 2H), 2.87 (t, J = 8.2 Hz, 2H), 1.17 (t, J = 7.2 Hz, 3H). ^{13}C NMR (126 MHz, $CDCl_3$) δ 155.9, 153.9, 124.7, 122.6, 103.7, 95.7, 77.4, 77.2, 76.9, 52.9, 43.1, 27.8, 11.9.

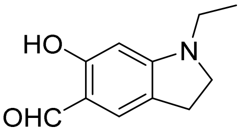

1-ethyl-6-hydroxyindoline-5-carbaldehyde (11d). $POCl_3$ (0.40 mL, 4.25 mmol, 1.2 eq.) was added to 5 mL anhydrous DMF under Ar protection. The solution was cooled to 0 °C and stirred for 30 min followed by the addition of **12d** (577 mg, 3.54 mmol) in 5 mL dry DMF. The temperature was allowed to rise to 90 °C and the reaction was kept stirring for 5 h. After cooled to room temperature, the reaction was quenched upon addition of 30 mL iced water and the pH was adjusted to neutral with solid $NaHCO_3$. The aqueous phase was extracted with DCM (50 mL ×3) and the combined organic layers were dried over Na_2SO_4, filtered through a pad of silica. The solvent was removed in vacuo to yield **11d** as brown oil, which slowly crystalized to solid (444 mg, 66%). ^{1}H NMR (400 MHz, $CDCl_3$) δ 12.28 (s, 1H), 9.39 (s, 1H), 6.98 (s, 1H), 5.82 (s, 1H), 3.63 (t, J = 8.3 Hz, 2H), 3.29 (q, J = 7.2 Hz, 2H), 2.98 (t, J = 8.3 Hz, 2H), 1.21 (t, J = 7.2 Hz, 3H). ^{13}C NMR (101 MHz, $CDCl_3$) δ 191.0, 166.4, 158.5, 127.8, 122.3, 111.3, 91.6, 77.4, 77.1, 76.8, 51.4, 40.9, 26.2, 11.6.

Synthesis of O-cored MARS model compounds.

General methods to synthesize O-cored pyronin intermediates and 9-cyanopyronins are described as following:

Method A: 4-(dialkylamino)-2-hydroxybenzaldehyde (**11a-c**, 0.1 mmol, 1 eq.) and 3-dialkylaminophenol (**12a-e**, 0.1 mmol, 1 eq.) were placed in a 25 mL round-bottom flask followed by the addition of 2.0 mL protonic acids (MsOH or 85% H_3PO_4). The flask was sealed, and the reaction was heated to 90 °C and kept stirring overnight. After cooled to room temperature, to the reaction was added 20 mL saturated brine. The aqueous phase was extracted with DCM (20 mL×3). The combined organic layers were washed with saturated brine, dried over Na_2SO_4 and concentrated. The crude product was purified with silica gel flash chromatograph (MeOH:DCM, 1:15, v/v) to afford desired pyronins (**13a-i**) as purple solid.

Method B: Freshly obtained pyronin (**13a-i**, 25 μmol, 1 eq.) was dissolved in a mixed solvent of 5 mL acetonitrile and 1 mL H_2O. To the vial was slowly added 0.5 mL 0.1 M KCN aqueous solution (50 μmol, 2 eq.). The reaction was stirred at room temperature for 30 min monitored by TLC until the strong magenta color disappeared. The intermediate was sensitive to oxidation and quickly treated with 0.5 mL 0.5 M $FeCl_3$ in 1 N HCl (0.25 mmol, 10 eq.) solution. After stirred for additional one hour, the reaction was quenched upon addition of 20 mL saturated brine. The aqueous phase was extracted with DCM (20 mL×3). The combined organic layers were washed with saturated brine and dried over Na_2SO_4. The solvent was removed in vacuo and the residue was purified via

silica gel flash chromatography (MeOH:DCM, 1:15, v/v) to give desired 9-cyanopyronins as dark blue solid.

Supplementary Figure 10. Syntheses of five pyronin intermediates (**13a-e**) and corresponding 9-cyanopyronin model compounds (**1-5, 14**) with different number of expanded rings.

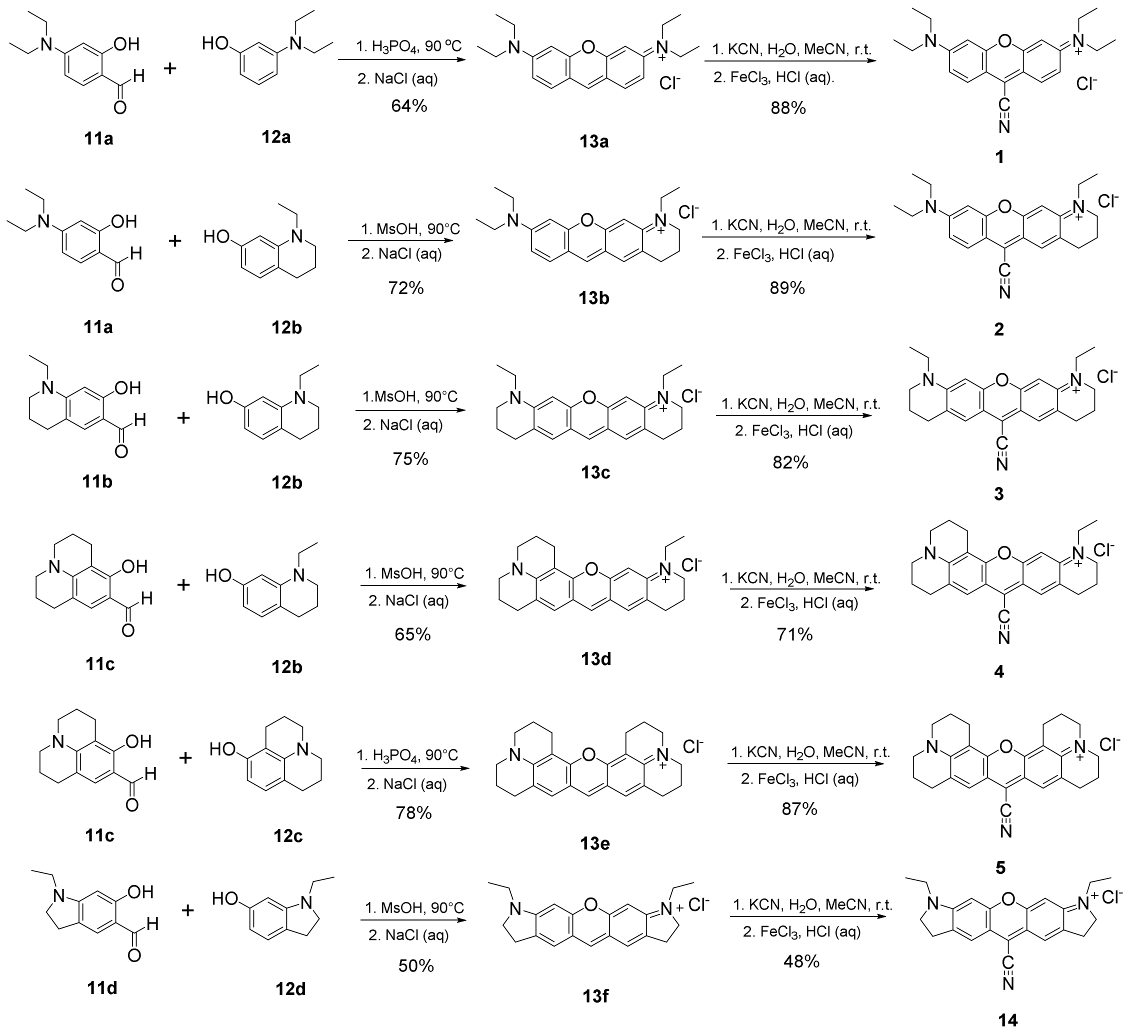

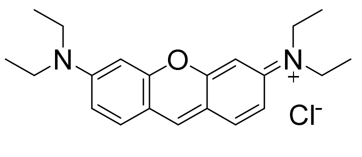

Pyronin intermediate 13a. *Method A* was used to synthesize molecule **13a**. **11a** (11.7 mg, 61 µmol) and **12a** (10.0 mg, 61 µmol) were treated with phosphoric acid to obtain **13a** as purple solid (14.0 mg, 64%). ^{1}H NMR (400 MHz, MeOD) δ 8.58 (s, 1H), 7.82 (d, *J* = 9.4 Hz, 2H), 7.20 (dd, *J* = 9.3, 2.4 Hz, 2H), 6.95 (d, *J* = 1.8 Hz, 2H), 3.73 (q, *J* = 7.1 Hz, 8H), 1.35 (t, *J* = 7.2 Hz, 12H).^{13}C NMR (101 MHz, MeOD) δ 159.7, 157.7, 146.9, 134.6, 115.6, 115.5, 97.2, 46.9, 12.8. HRMS (ESI+) m/z Calcd. for $C_{21}H_{27}N_2O^+$ [M]+: 323.2131. Found: 323.2142

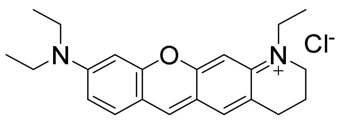

Pyronin intermediate 13b. 13b was obtained from **11a** (11.0 mg, 56 µmol) and **12b** (10.0 mg, 56 µmol) using *method A* with methenesulfonic acid (MsOH). Purification using silica gel chromatography yielded **13b** as purple solid (15.0 mg, 72%). ^{1}H NMR (400 MHz, MeOD) δ 8.42 (s, 1H), 7.76 (d, *J* = 9.3 Hz, 1H), 7.47 (s, 1H), 7.14 (dd, *J* = 9.3, 2.4 Hz, 1H), 6.88 (d, *J* = 2.1 Hz, 1H), 6.87 (s, 1H), 3.73 – 3.61 (m, 8H), 2.89 (t, *J* = 6.3 Hz, 2H), 2.03 (p, *J* = 6.1 Hz, 2H), 1.37 – 1.30 (m, 9H). ^{13}C NMR (101 MHz, MeOD) δ 159.2, 159.1, 157.0, 156.0, 145.6, 134.1, 131.3, 127.2, 116.0, 115.1, 115.1, 97.1, 96.0, 50.7, 46.7, 39.5, 28.3, 22.0, 12.8, 11.4. HRMS (ESI+) m/z Calcd. for $C_{22}H_{27}N_2O^+$ [M]$^+$: 335.2123. Found: 335.2143

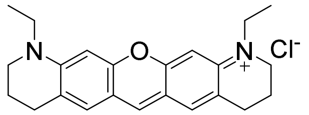

Pyronin intermediate 13c. 13c was obtained from **11b** (11.5 mg, 56 µmol) and **12b** (10.0 mg, 56 µmol) using *method A* with MsOH. Purification with silica gel chromatography afforded **13c** as purple solid (16.1 mg, 75%). ^{1}H NMR (400 MHz, MeOD) δ 8.31 (s, 1H), 7.44 (s, 2H), 6.84 (s, 2H), 3.70 – 3.59 (m, 8H), 2.92 – 2.85 (m, 4H), 2.02 (p, *J* = 6.0 Hz, 4H), 1.33 (t, *J* = 7.2 Hz, 6H). ^{13}C NMR (101 MHz, MeOD) δ 158.7, 155.4, 144.6, 130.9, 126.9, 115.6, 95.8, 50.5, 48.0, 28.4, 22.0, 11.3. HRMS (ESI+) m/z Calcd. for $C_{23}H_{27}N_2O^+$ [M]$^+$: 347.2123. Found: 347.2144

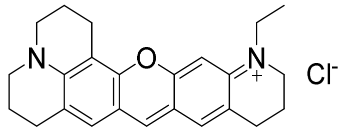

Pyronin intermediate 13d. 13d was obtained from **11c** (12.1 mg, 56 µmol) and **12b** (10.0 mg, 56 µmol) using *method A* with MsOH. Purification with silica gel chromatography yielded **13d** as purple solid (14.3 mg, 65%). ^{1}H NMR (400 MHz, MeOD) δ 8.22 (s, 1H), 7.41 (s, 1H), 7.32 (s, 1H), 6.84 (s, 1H), 3.69 – 3.55 (m, 8H), 3.00 (t, *J* = 6.4 Hz, 2H), 2.92 – 2.86 (m, 4H), 2.13 – 1.98 (m, 6H), 1.33 (t, *J* = 7.2 Hz, 3H). ^{13}C NMR (101 MHz, MeOD) δ 158.6, 154.9, 153.9, 153.3, 144.2, 130.8, 129.5, 126.4, 125.8, 115.7, 114.8, 106.4, 95.8, 52.1, 51.6, 50.4, 47.8, 28.4, 28.4, 22.1, 21.7, 20.7, 20.7, 11.3. HRMS (ESI+) m/z Calcd. for $C_{24}H_{27}N_2O^+$ [M]$^+$: 359.2123. Found: 359.2143

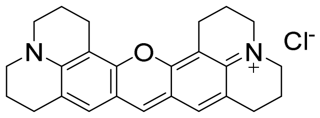

Pyronin intermediate 13e. 13e was obatained from **11c** (434 mg, 2.0 mmol) and **12c** (378 mg, 2.0 mmol) following *method A*. Purification with silica gel chromatography yielded **13e** (637 mg, 78%) as purple crystal. ^{1}H NMR (500 MHz, MeOD) δ 8.18 (s, 1H), 7.32 (s, 2H), 3.60 – 3.53 (m, 8H), 3.00 (t, *J* = 6.4 Hz, 4H), 2.92 – 2.86 (m, 4H), 2.09 (p, *J* = 6.4 Hz, 4H), 2.04 (p, *J* = 6.2 Hz, 4H).

^{13}C NMR (126 MHz, MeOD) δ 153.8, 152.9, 144.1, 129.2, 125.3, 115.0, 106.5, 52.0, 51.5, 28.5, 21.8, 20.8, 20.8. HRMS (ESI+) m/z Calcd. for $C_{25}H_{27}N_2O^+$ $[M]^+$: 371.2123. Found: 371.2143

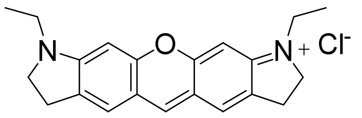

Pyronin intermediate 13f. 13f was obtained from **11d** (34 mg, 0.18 mmol) and **12d** (29 mg, 0.18 mmol) following method A. Purification with silica gel chromatography yielded **13f** as purple solid (32 mg, 50%). ^1H NMR (400 MHz, MeOD) δ 8.18 (s, 1H), 7.36 (s, 2H), 6.57 (s, 2H), 3.91 (t, *J* = 7.7 Hz, 4H), 3.54 (q, *J* = 7.1 Hz, 4H), 3.19 (t, *J* = 7.7 Hz, 4H), 1.29 (t, *J* = 7.2 Hz, 6H). ^{13}C NMR (101 MHz, MeOD) δ 160.9, 160.7, 143.7, 135.2, 125.9, 116.4, 91.4, 53.0, 42.3, 26.9, 11.9.

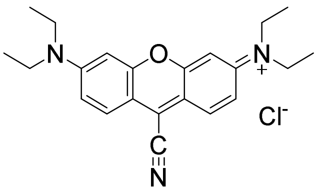

MARS2241 (1). *Method B* was used to synthesize compound **1**. **13a** (8.8 mg, 24 μmol) was treated with KCN and $FeCl_3$ sequentially to afford **1** as dark blue film (8.1 mg, 88%). ^1H NMR (500 MHz, MeOD) δ 7.95 (d, *J* = 9.5 Hz, 2H), 7.37 (dd, *J* = 9.5, 2.4 Hz, 2H), 7.03 (d, *J* = 2.4 Hz, 2H), 3.79 (q, *J* = 7.2 Hz, 8H), 1.37 (t, *J* = 7.2 Hz, 12H). ^{13}C NMR (126 MHz, MeOD) δ 157.4, 156.6, 129.9, 122.4, 116.1, 114.2, 111.9, 96.7, 46.1, 11.5. HRMS (ESI+) m/z Calcd. for $C_{22}H_{26}N_3O^+$ $[M]^+$: 348.2076. Found: 348.2077

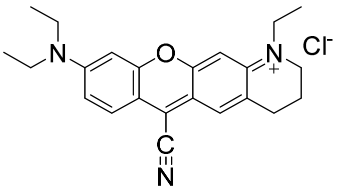

MARS2240 (2). 2 was obtained from **13b** (6.7 mg, 18 μmol) using *method B* as described above. After silica gel chromatograph purification, **2** was obtained as dark blue film (6.3 mg, 89%). ^1H NMR (500 MHz, MeOD) δ 7.88 (d, *J* = 9.4 Hz, 1H), 7.61 (t, *J* = 1.3 Hz, 1H), 7.30 (dd, *J* = 9.4, 2.4 Hz, 1H), 6.99 (s, 1H), 6.96 (d, *J* = 2.4 Hz, 1H), 3.79 – 3.69 (m, 10H), 2.97 (t, *J* = 5.8 Hz, 2H), 2.06 (p, *J* = 6.2 Hz, 2H), 1.38 – 1.32 (m, 9H). ^{13}C NMR (126 MHz, MeOD) δ 158.5, 158.2, 157.2, 156.6, 130.8, 130.1, 127.9, 122.3, 116.8, 116.7, 114.6, 113.4, 97.9, 97.1, 51.3, 47.2, 28.3, 21.8, 12.9, 11.6. HRMS (ESI+) m/z Calcd. for $C_{23}H_{26}N_3O^+$ $[M]^+$: 360.2076. Found: 360.2060

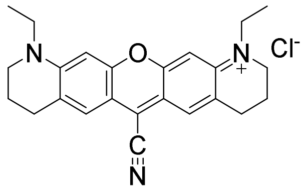

MARS2239 (3). 3 was obtained from **13c** (7.1 mg, 18 µmol) using *method B* as described above. Purificiation with silica gel chromatography afforded **3** as dark blue solid (6.0 mg, 82%). ^{1}H NMR (500 MHz, MeOD) δ 7.57 (s, 2H), 6.95 (s, 2H), 3.72 (dt, *J* = 13.2, 6.5 Hz, 8H), 2.97 (t, *J* = 6.1 Hz, 4H), 2.07 (p, *J* = 6.1 Hz, 4H), 1.36 (t, *J* = 7.1 Hz, 6H). ^{13}C NMR (126 MHz, MeOD) δ 156.4, 154.3, 128.0, 126.1, 119.7, 114.1, 112.1, 95.4, 49.6, 47.2, 27.0, 20.4, 10.1. HRMS (ESI+) m/z Calcd. for $C_{24}H_{26}N_3O^+$ [M]$^+$: 372.2076. Found: 372.2079

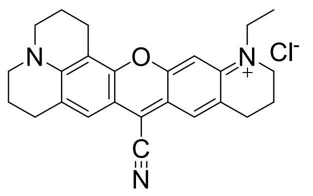

MARS2238 (4). 4 was obtained from **13d** (8.3 mg, 21 µmol) following method B as described before. Purification with silica gel chromatography afforded **4** as dark blue film (6.3 mg, 71%). ^{1}H NMR (500 MHz, MeOD) δ 7.52 (s, 1H), 7.46 (s, 1H), 6.94 (s, 1H), 3.75 – 3.62 (m, 8H), 3.01 (t, *J* = 6.3 Hz, 2H), 2.99 – 2.93 (m, 4H), 2.08 (dq, *J* = 15.7, 5.7 Hz, 8H), 1.35 (t, *J* = 7.1 Hz, 3H). ^{13}C NMR (126 MHz, MeOD) δ 157.6, 155.2, 154.0, 152.7, 128.6, 127.4, 126.1, 120.5, 116.2, 114.2, 113.7, 107.8, 96.7, 52.7, 52.2, 50.8, 48.4, 28.4, 28.4, 21.9, 21.5, 20.7, 20.4, 11.5. HRMS (ESI+) m/z Calcd. for $C_{25}H_{26}N_3O^+$ [M]$^+$: 384.2076. Found: 384.2090

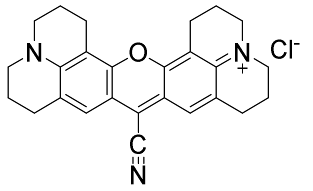

MARS2237 (5). 5 was obtained from **13e** (16.0 mg, 39 µmol) following *method B*. After silica gel chromatography, **5** was afforded as dark blue solid (14.7 mg, 87%). ^{1}H NMR (500 MHz, MeOD) δ 7.38 (s, 2H), 3.69 – 3.62 (m, 8H), 2.99 – 2.92 (m, 8H), 2.15 – 2.05 (m, 8H). ^{13}C NMR (126 MHz, MeOD) δ 152.0, 151.0, 126.4, 124.4, 118.5, 113.3, 112.4, 106.4, 51.1, 50.6, 27.0, 20.2, 19.4, 19.1.

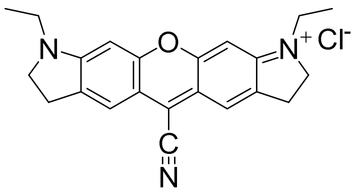

MARS2239P (14). 14 was obtained from **13f** (7.8 mg, 22 µmol) using *method B* as described above. Yield: 4.0 mg, 48%. ^{1}H NMR (400 MHz, MeOD) δ 7.58 (s, 2H), 6.74 (s, 2H), 4.05 (t, *J* = 7.0 Hz, 4H), 3.67 (q, *J* = 7.0 Hz, 4H), 3.31 (t, *J* = 7.1 Hz, 4H), 1.35 (t, *J* = 7.1 Hz, 6H), ^{13}C NMR (101 MHz, MeOD) δ 161.1, 160.1, 138.1, 122.7, 120.4, 116.4, 113.8, 92.4, 53.6, 42.7, 27.1, 12.1. HRMS (ESI+) m/z Calcd. for $C_{22}H_{22}N_3O^+$ [M]$^+$: 344.1763. Found: 344.1789

Synthesis of O-cored MARS NHS esters.

Supplementary Figure 11. Syntheses of pyronin intermediates bearing a carboxylic acid side chain. Conditions: (1) excessive MsOH, 90 °C, 4 hrs. (2) adding water, 90 °C, additional 4 hrs.

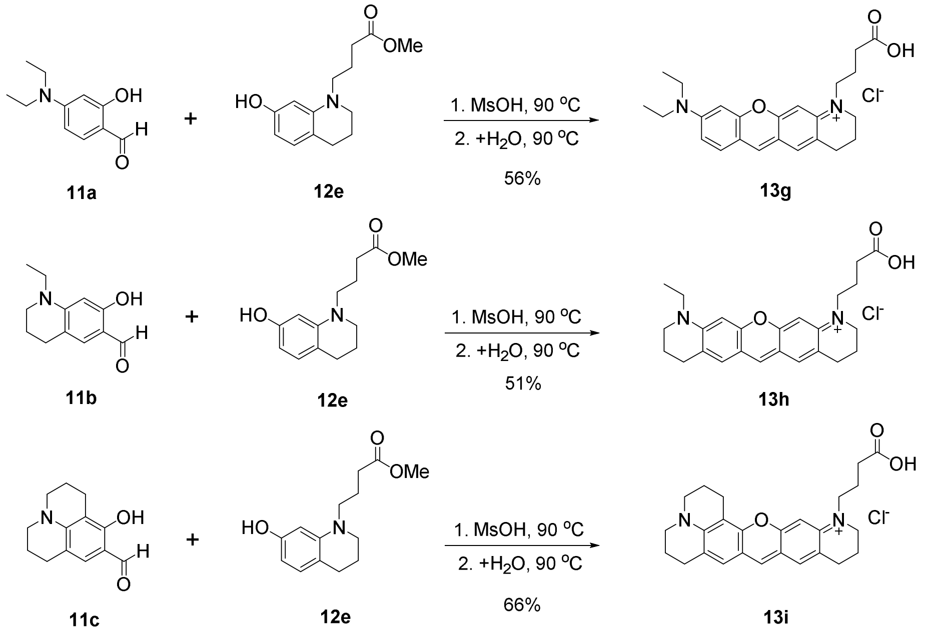

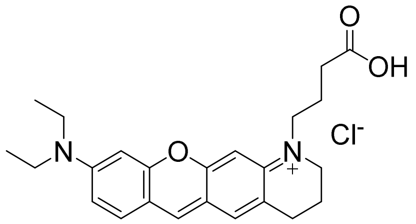

Pyronin intermediate 13g. To a mixture of **11a** (135 mg, 0.70 mmol) and **12e** (175 mg, 0.70 mmol) was added 7 mL methanesulfonic acid. The solution was heated to 90 °C and stirred overnight. 7 mL DI water was added into the reaction and the mixture was further stirred for 4h at 90 °C. After cooled down to r.t., the mixture was poured into 20 g ice and diluted with 40 mL brine. The aqueous phase was extracted with DCM (40 mL ×5). The organic phase was combined, washed with brine, dried over Na_2SO_4 and concentrated. The crude product was purified through silica gel chromatography (MeOH: DCM 15:1, v/v) to give **13g** as dark purple film (168 mg, 56%). ^{1}H NMR (400 MHz, MeOD) δ 8.47 (s, 1H), 7.79 (d, *J* = 9.2 Hz, 1H), 7.51 (s, 1H), 7.17 (dd, *J* = 9.3, 2.4 Hz, 1H), 7.04 (s, 1H), 6.91 (d, *J* = 2.5 Hz, 1H), 3.78 – 3.60 (m, 8H), 2.92 (t, *J* = 6.3 Hz, 2H), 2.51 (t, *J* = 6.7 Hz, 2H), 2.04 (dq, *J* = 12.9, 6.6 Hz, 4H), 1.34 (t, *J* = 7.1 Hz, 6H). ^{13}C NMR (101 MHz, MeOD) δ 176.4, 159.3, 159.2, 157.1, 156.4, 145.8, 134.2, 131.3, 127.2, 115.3, 115.2, 97.1, 96.4, 52.8, 51.5, 46.8, 39.5, 31.4, 28.4, 22.2, 21.9, 12.8. HRMS (ESI+) m/z Calcd. for $C_{24}H_{29}N_2O_3^+$ [M]$^+$: 393.2178. Found: 393.2183

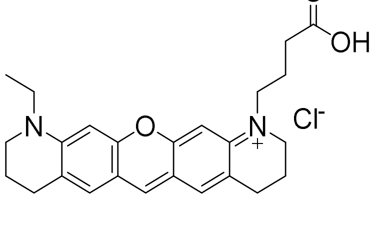

Pyronin intermediate 13h. 13h was obtained from **11b** (41 mg, 0.2 mmol) and **12e** (50 mg, 0.2 mmol) following the same protocol used for **13g**. Yield: 45 mg, 51%. ^{1}H NMR (400 MHz, MeOD) δ 8.33 (s, 1H), 7.45 (s, 2H), 7.02 (s, 1H), 6.84 (s, 1H), 3.65 (m, 8H), 2.92 – 2.86 (m, 4H), 2.37 (t, *J* = 6.9 Hz, 2H), 2.05 – 1.99 (m, 6H), 1.33 (t, *J* = 7.1 Hz, 3H). ^{13}C NMR (101 MHz, MeOD) δ 158.7, 158.6, 155.6, 155.3, 144.6, 130.9, 126.8, 126.7, 115.6, 96.3, 95.7, 53.1, 51.3, 50.5, 48.0, 33.8, 30.8, 28.4, 23.0, 22.0, 22.0, 11.3. HRMS (ESI+) m/z Calcd. for $C_{25}H_{29}N_2O_3^+$ [M]$^+$: 405.2178. Found: 405.2192

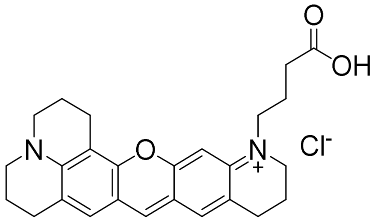

Pyronin intermediate 13i. 13i was obtained from **11c** (43 mg, 0.2 mmol) and **12e** (50 mg, 0.2 mmol) following the same protocol used for **13g**. Yield: 60 mg, 66%. ^{1}H NMR (400 MHz, MeOD) δ 8.22 (s, 1H), 7.40 (d, *J* = 1.2 Hz, 1H), 7.33 (d, *J* = 1.3 Hz, 1H), 6.97 (s, 1H), 3.65 – 3.52 (m, 8H), 3.02 (t, *J* = 6.3 Hz, 2H), 2.90 – 2.85 (m, 4H), 2.33 (t, *J* = 7.0 Hz, 2H), 2.10 – 1.97 (m, 8H). ^{13}C NMR (126 MHz, MeOD) δ 177.1, 158.4, 155.1, 153.8, 153.3, 144.2, 130.8, 129.5, 126.2, 125.8, 115.8, 114.8, 106.4, 96.3, 52.6, 52.1, 51.6, 51.2, 28.4, 22.3, 22.0, 21.7, 20.7. HRMS (ESI+) m/z Calcd. for $C_{26}H_{29}N_2O_3^+$ [M]$^+$: 417.2178. Found: 417.2179

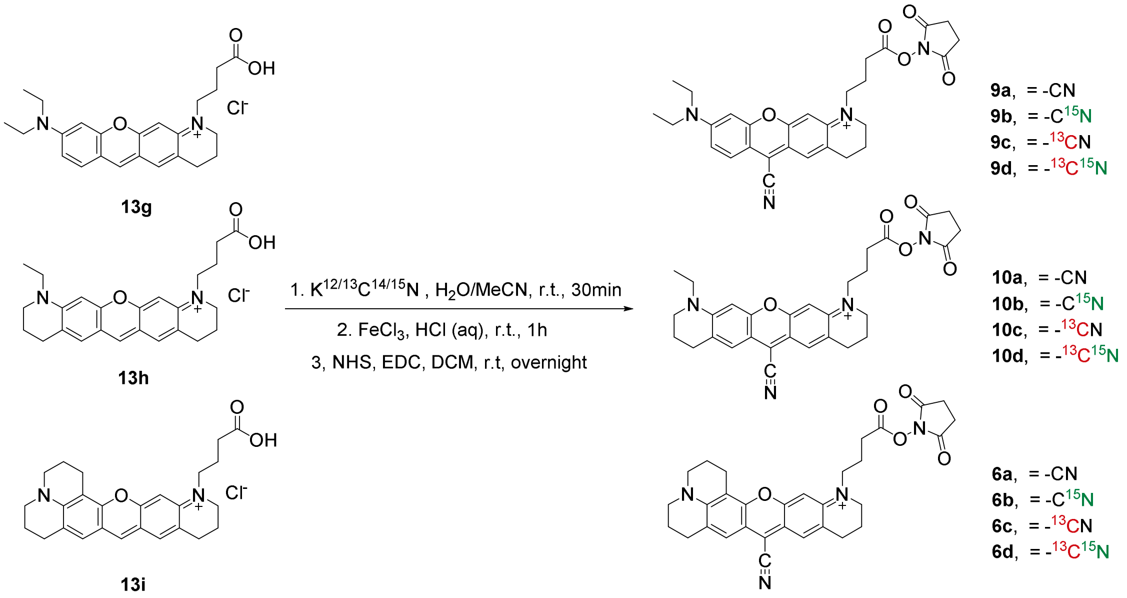

Supplementary Figure 12. Syntheses of 3 sets of O-cored MARS NHS esters each containing 4 nitrile isotopologues. The first two steps followed aforementioned *method B*. Counter ions for all NHS esters purified via HPLC are TFA anion.

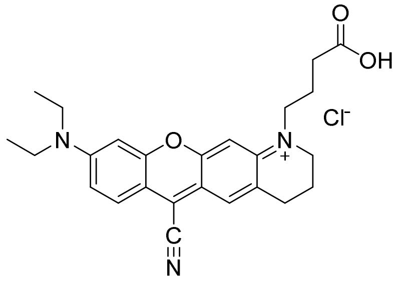

MARS2240-COOH (15). Following *method B*, freshly prepared **13g** (16 mg, 33 μmol) was dissolved in a mixture of 5 mL acetonitrile and 1 mL water. To the solution was added 0.66 mL 0.1 M KCN (66 μmol, 2 eq.) aqueous solution dropwise via syringe. The reaction was stirred at r.t. for 30 min until reactant was fully consumed (solution turned into light blue). 0.66 mL 0.5 $FeCl_3$ (in 1 N HCl solution, 0.66 mmol, 10 eq.) was then added to the reaction, which was further stirred for 1h. The reaction was diluted with 20 mL saturated brine and extracted with DCM (20 mL ×3). The combined organic layers were dried over Na_2SO_4 and concentrated to afford crude product, which was further purified with chromatography (MeOH:DCM 30:1-10:1) to get **15** as dark blue film (12 mg, 81%). ^1H NMR (400 MHz, MeOD) δ 7.89 (d, *J* = 9.4 Hz, 1H), 7.62 (d, *J* = 1.2 Hz, 1H), 7.31 (dd, *J* = 9.5, 2.4 Hz, 1H), 7.14 (s, 1H), 6.95 (d, *J* = 2.4 Hz, 1H), 3.79 – 3.69 (m, 8H), 2.98 (t, *J* = 5.7 Hz, 2H), 2.44 (t, *J* = 6.8 Hz, 2H), 2.11 – 1.99 (m, 4H), 1.34 (t, *J* = 7.1 Hz, 6H). ^{13}C NMR (101 MHz, MeOD) δ 175.3, 158.4, 158.3, 157.2, 156.9, 130.8, 130.0, 127.9, 122.4, 116.9, 116.6, 114.7, 113.4, 97.9, 97.6, 53.7, 52.1, 47.3, 28.3, 23.0, 21.8, 12.9.

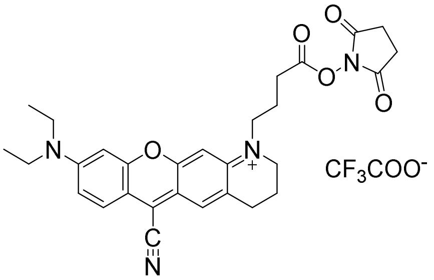

MARS2241-NHS (9a). A round-bottom flask was filled with **15** (12 mg, 26 μmol), N-Hydroxysuccinimide (NHS, 8.9 mg, 78 μmol, 3 eq.), and N-(3-Dimethylaminopropyl)-N′-ethylcarbodiimide hydrochloride (EDC, 19.8 mg, 104 μmol, 4 eq.). 3 mL Anhydrous DCM was added into the flask to dissolve the solid. The solution was stirred overnight at r.t. and evaporated upon vacuum. The residue was dissolved in 2 mL acetonitrile and purified with HPLC (eluent: MeCN/H_2O, TFA, 20%-90%) to afford **9a** as dark blue film (TFA salt, 11.7 mg, 72%). ^1H NMR (400 MHz, MeOD) δ 7.89 (d, *J* = 9.4 Hz, 1H), 7.61 (s, 1H), 7.32 (dd, *J* = 9.5, 2.5 Hz, 1H), 7.03 (s, 1H), 6.97 (d, *J* = 2.4 Hz, 1H), 3.84 – 3.69 (m, 8H), 2.98 (t, *J* = 6.3 Hz, 2H), 2.91 – 2.85 (m, 6H), 2.18 (p, *J* = 6.9 Hz, 2H), 2.07 (p, *J* = 6.5 Hz, 2H), 1.34 (t, *J* = 7.1 Hz, 6H). ^{13}C NMR (101 MHz, MeOD) δ 170.4, 168.7, 157.0, 157.0, 156.0, 155.4, 129.5, 128.4, 126.6, 121.2, 115.8, 115.0, 113.7, 112.0, 96.5, 95.9, 51.3, 50.6, 45.9, 27.3, 26.9, 25.1, 20.9, 20.3, 11.5. HRMS (ESI+) m/z Calcd. for $C_{29}H_{31}N_4O_5^+$ $[M]^+$: 515.2294. Found: 515.2299

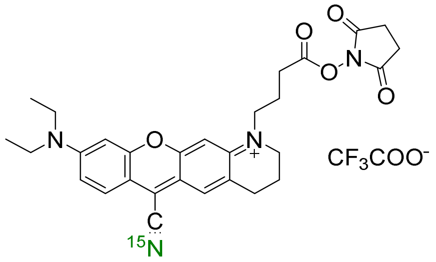

MARS2212-NHS (9b). 9b was obtained from **13g** following the same protocols as for **15** and **9a**. KCN was replaced by $KC^{15}N$. ^{1}H NMR (400 MHz, MeOD) δ 7.91 (d, *J* = 9.5 Hz, 1H), 7.64 (s, 1H), 7.33 (dd, *J* = 9.5, 2.4 Hz, 1H), 7.05 (s, 1H), 6.99 (d, *J* = 2.4 Hz, 1H), 3.84 – 3.69 (m, 8H), 2.98 (t, *J* = 5.8 Hz, 2H), 2.88 (d, *J* = 4.5 Hz, 6H), 2.19 (dt, *J* = 14.8, 6.8 Hz, 2H), 2.07 (p, *J* = 6.3, 5.9 Hz, 2H), 1.34 (t, *J* = 7.1 Hz, 6H). ^{13}C NMR (101 MHz, MeOD) δ 171.8, 170.1, 158.5, 158.4, 157.4, 156.8, 130.9, 129.9, 128.0, 122.7 (d, *J* = 2.8 Hz), 117.2, 116.4, 115.1, 113.4 (d, *J* = 17.2 Hz), 97.9, 97.3, 52.7, 52.0, 47.3, 28.7, 28.3, 26.5, 22.3, 21.7, 12.9. HRMS (ESI+) m/z Calcd. for $C_{29}H_{31}N_3{}^{15}NO_5{}^+$ $[M]^+$: 516.2265. Found: 516.2266

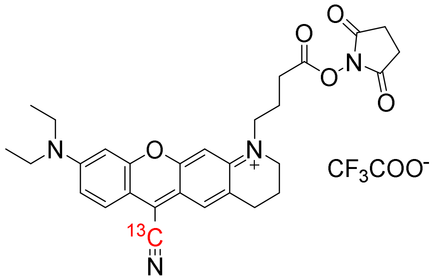

MARS2186-NHS (9c). 9c was obtained from **13g** following the same protocols as for **15** and **9a**. ^{1}H NMR (400 MHz, MeOD) δ 7.88 (d, *J* = 9.5 Hz, 1H), 7.60 (s, 1H), 7.32 (dd, *J* = 9.5, 2.5 Hz, 1H), 7.02 (s, 1H), 6.97 (d, *J* = 2.4 Hz, 1H), 3.85 – 3.69 (m, 8H), 3.01 – 2.94 (m, 2H), 2.88 (d, *J* = 3.3 Hz, 6H), 2.18 (dt, *J* = 14.6, 6.9 Hz, 2H), 2.07 (p, *J* = 6.5 Hz, 2H), 1.35 (t, *J* = 7.1 Hz, 6H). ^{13}C NMR (101 MHz, MeOD) δ 171.8, 170.1, 158.4 (d, *J* = 4.5 Hz), 158.3 (d, *J* = 4.2 Hz), 157.4, 156.8, 130.9 (d, *J* = 3.1 Hz), 129.9, 128.0 (d, *J* = 3.2 Hz), 122.6 (d, *J* = 81.9 Hz), 117.2, 116.4 (d, *J* = 2.1 Hz), 115.1 (d, *J* = 2.1 Hz), 113.4, 97.9, 97.3, 52.7, 52.0, 47.3, 28.7, 28.3, 26.5, 22.3, 21.7, 12.9. HRMS (ESI+) m/z Calcd. for $C_{28}{}^{13}CH_{31}N_4O_5{}^+$ $[M]^+$: 516.2328. Found: 516.2325

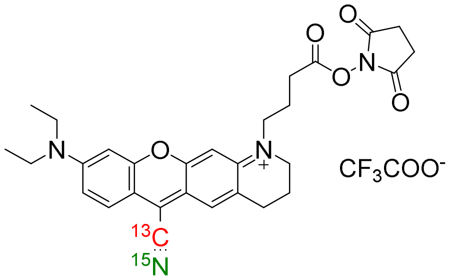

MARS2157-NHS (9d). 9d was obtained from **13g** following the same protocols as for **15** and **9a**. ^{1}H NMR (400 MHz, MeOD) δ 7.90 (d, *J* = 9.5 Hz, 1H), 7.62 (s, 1H), 7.34 (dd, *J* = 9.5, 2.4 Hz, 1H), 7.04 (s, 1H), 6.99 (d, *J* = 2.2 Hz, 1H), 3.85 – 3.71 (m, 8H), 2.99 (t, *J* = 5.9 Hz, 2H), 2.90 (d, *J* = 3.0 Hz, 6H), 2.20 (p, *J* = 6.9 Hz, 2H), 2.09 (p, *J* = 6.4 Hz, 2H), 1.37 (t, *J* = 7.1 Hz, 6H). ^{13}C NMR (101 MHz, MeOD) δ 171.8, 170.1, 158.4 (d, *J* = 4.7 Hz), 158.3 (d, *J* = 4.7 Hz), 157.4, 156.8, 130.9 (d, *J* = 3.0 Hz), 129.9, 128.0 (d, *J* = 3.2 Hz), 122.6 (dd, *J* = 81.9, 3.0 Hz), 117.2, 116.4 (d, *J* = 1.8 Hz), 115.1 (d, *J* = 2.3 Hz), 113.4 (d, *J* = 16.9 Hz), 97.9, 97.3, 52.7, 52.0, 47.3, 28.7, 28.3, 26.5, 22.3, 21.7, 12.9. HRMS (ESI+) m/z Calcd. for $C_{28}{}^{13}CH_{31}N_{3}{}^{15}NO_{5}^{+}$ [M]$^{+}$: 517.2299. Found: 517.2300.

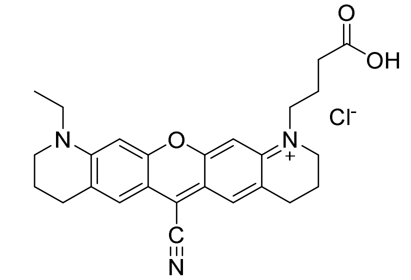

MARS2239-COOH (16). 16 was obtained from **13h** (17 mg, 38 µmol) following *method B*. Yield: 12 mg, 69%. ^{1}H NMR (500 MHz, MeOD) δ 7.53 (s, 1H), 7.52 (s, 1H), 7.06 (s, 1H), 6.91 (s, 1H), 3.70 (dp, *J* = 15.6, 8.2, 7.8 Hz, 8H), 2.95 (t, *J* = 5.9 Hz, 4H), 2.46 (t, *J* = 6.3 Hz, 2H), 2.09 – 1.98 (m, 6H), 1.35 (t, *J* = 7.1 Hz, 3H). ^{13}C NMR (126 MHz, MeOD) δ 178.2, 157.8, 157.7, 156.0, 155.8, 129.5, 129.3, 127.5, 127.5, 121.0, 115.7, 115.4, 113.5, 97.2, 96.8, 53.3, 51.8, 51.1, 32.2, 28.4, 22.7, 21.8, 21.8, 11.6.

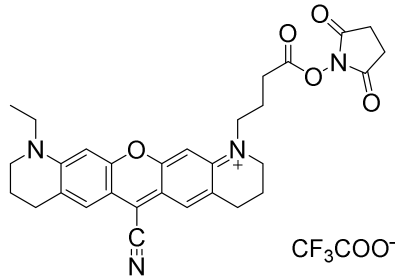

MARS2239-NHS (10a). 10a was obtained from **16** (10 mg, 21 µmol) following the same protocol for **9a**. Yield: 8.8 mg, 65%. ^{1}H NMR (400 MHz, MeOD) δ 7.62 – 7.54 (m, 2H), 7.00 (s, 1H), 6.96 (s, 1H), 3.81 – 3.66 (m, 8H), 3.02 – 2.92 (m, 4H), 2.87 (d, *J* = 1.7 Hz, 6H), 2.17 (p, *J* = 6.9 Hz, 2H), 2.06 (p, *J* = 5.9 Hz, 4H), 1.35 (t, *J* = 7.2 Hz, 3H). ^{13}C NMR (126 MHz, MeOD) δ 171.8, 170.2, 158.0, 157.8, 156.0, 156.0, 129.7, 129.2, 127.7, 127.6, 121.4, 116.1, 115.3, 113.5, 97.0, 96.9, 52.4, 51.8, 51.1, 28.7, 28.4, 26.5, 22.2, 21.8, 21.8, 11.6. HRMS (ESI+) m/z Calcd. for $C_{30}H_{31}N_{4}O_{5}^{+}$ [M]$^{+}$: 527.2289. Found: 527.2284.

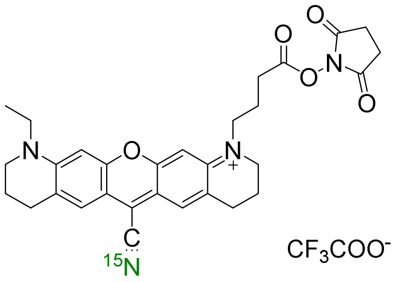

MARS2211-NHS (10b). ^{1}H NMR (400 MHz, MeOD) δ 7.58 (s, 2H), 6.99 (s, 1H), 6.96 (s, 1H), 3.80 – 3.66 (m, 8H), 2.96 (t, *J* = 6.3 Hz, 4H), 2.91 – 2.83 (m, 6H), 2.17 (p, *J* = 7.0 Hz, 2H), 2.06 (p, *J* = 6.2 Hz, 4H), 1.35 (t, *J* = 7.1 Hz, 3H).^{13}C NMR (101 MHz, Methanol-d_4) δ 171.8 , 170.2 , 158.0 , 157.8 , 156.0 , 156.0 , 129.8 , 129.2 , 127.7 , 127.6 , 121.4 (d, *J* = 2.9 Hz), 116.1 , 115.3 , 113.5 (d, *J* = 16.9 Hz), 97.0 , 96.9 , 52.4 , 51.8 , 51.1 , 28.7 , 28.4 , 26.5 , 22.2 , 21.8 , 21.8 , 11.6 . HRMS (ESI+) m/z Calcd. for $C_{30}H_{31}N_3{}^{15}NO_5{}^+$ $[M]^+$: 528.2265. Found: 528.2264.

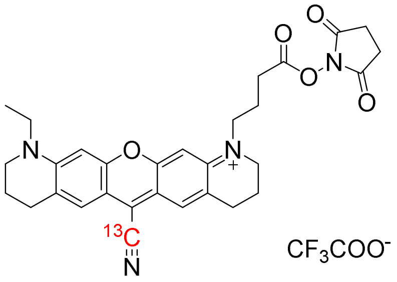

MARS2185-NHS (10c). ^{1}H NMR (400 MHz, MeOD) δ 7.57 (s, 2H), 6.98 (s, 1H), 6.95 (s, 1H), 3.83 – 3.62 (m, 8H), 2.96 (t, *J* = 6.3 Hz, 4H), 2.92 – 2.82 (m, 6H), 2.17 (p, *J* = 6.9 Hz, 2H), 2.06 (p, *J* = 6.2 Hz, 4H), 1.35 (t, *J* = 7.1 Hz, 3H). ^{13}C NMR (101 MHz, Methanol-d_4) δ 171.8 , 170.2 , 158.0 (d, *J* = 4.4 Hz), 157.8 (d, *J* = 4.9 Hz), 156.0 , 155.9 , 129.8 , 129.2 , 127.6 (d, *J* = 3.1 Hz), 127.5 (d, *J* = 3.3 Hz), 121.3 (d, *J* = 81.8 Hz), 116.1 (d, *J* = 1.3 Hz), 115.3 (d, *J* = 1.5 Hz), 113.5 , 97.0 , 96.9 , 52.4 , 51.8 , 51.1 , 28.7 , 28.4 , 26.5 , 22.2 , 21.8 , 21.8 , 11.6. HRMS (ESI+) m/z Calcd. for $C_{29}{}^{13}CH_{31}N_4O_5{}^+$ $[M]^+$: 528.2328. Found: 528.2332.

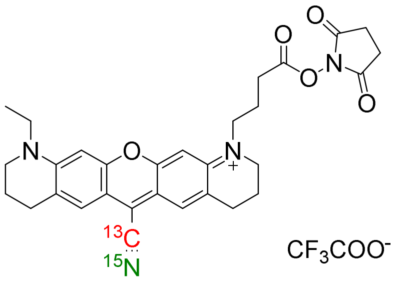

MARS2156-NHS (10d). ^{1}H NMR (400 MHz, MeOD) δ 7.57 (s, 2H), 6.98 (s, 1H), 6.95 (s, 1H), 3.82 – 3.60 (m, 8H), 2.96 (t, *J* = 6.2 Hz, 4H), 2.92 – 2.83 (m, 6H), 2.17 (p, *J* = 6.9 Hz, 2H), 2.06 (p, *J* = 6.2 Hz, 4H), 1.35 (t, *J* = 7.2 Hz, 3H). ^{13}C NMR (126 MHz, Methanol-d_4) δ 171.8 , 170.2 , 158.0 (d, *J* = 4.4 Hz), 157.8 (d, *J* = 4.4 Hz), 156.0 , 155.9 , 129.8 , 129.2 , 127.6 (d, *J* = 3.1 Hz), 127.5 (d, *J* = 3.3 Hz), 121.3 (dd, *J* = 81.8, 3.3 Hz), 116.1 (d, *J* = 1.9 Hz), 115.3 (d, *J* = 2.2 Hz), 113.5 (d, *J* = 16.9 Hz), 97.0 , 96.9 , 52.4 , 51.8 , 51.1 , 28.7 , 28.4 , 26.5 , 22.2 , 21.8 , 21.8 , 11.6.

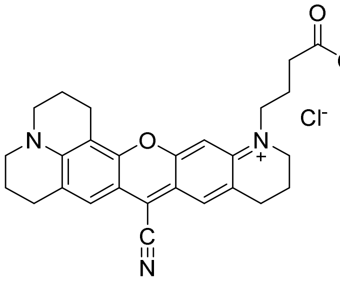

MARS2238-COOH (17). 17 was obtained from **13i** (12.2 mg, 27 μmol) following *method B*. Yield: 10.0 mg, 77%. ^{1}H NMR (500 MHz, Methanol-d_4) δ 7.42 (d, *J* = 1.3 Hz, 1H), 7.37 (d, *J* = 1.4 Hz, 1H), 7.03 (s, 1H), 3.69 (m, 6H), 3.65 – 3.59 (t, 2H), 2.99 – 2.90 (m, 6H), 2.48 (t, *J* = 6.7 Hz, 2H), 2.13 – 1.96 (m, 8H). ^{13}C NMR (126 MHz, MeOD) δ 175.1, 155.9, 154.1, 152.6, 151.1, 127.4, 127.1, 125.9, 124.6, 118.7, 114.8, 112.5, 112.2, 106.4, 95.8, 51.8, 51.3, 50.8, 50.2, 27.0, 21.2, 20.4, 20.1, 19.2, 19.0. HRMS (ESI+) m/z Calcd. for $C_{27}H_{28}N_3O_3^+$ [M]$^+$: 442.2131. Found: 442.2132

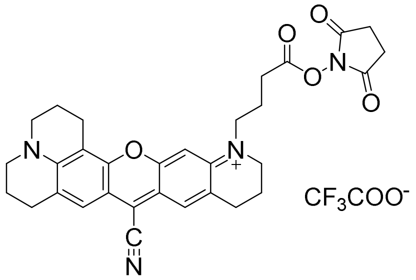

MARS2238-NHS (6a). 6a was obtained from **17** (10.0 mg, 21 μmol) following same method used to synthesize **9a**. Yield: 5.0 mg, 36%. ^{1}H NMR (400 MHz, Methanol-d_4) δ 7.51 (s, 1H), 7.46 (s, 1H), 7.02 (s, 1H), 3.73 (t, *J* = 7.3 Hz, 2H), 3.70 – 3.63 (m, 6H), 3.01 (t, *J* = 6.4 Hz, 2H), 2.98 – 2.92 (m, 4H), 2.88 (s, 4H), 2.85 (t, *J* = 6.9 Hz, 2H), 2.16 (p, *J* = 7.3 Hz, 2H), 2.11 – 2.01 (m, 6H). ^{13}C NMR (101 MHz, MeOD) δ 170.4, 168.6, 156.1, 154.0, 152.8, 127.6, 127.0, 126.1, 124.7, 119.3, 115.4, 112.6, 112.3, 106.5, 95.7, 51.3, 50.9, 50.1, 27.5, 27.0, 27.0, 25.2, 21.0, 20.4, 20.1, 19.2, 19.0. HRMS (ESI+) m/z Calcd. for $C_{31}H_{31}N_4O_5^+$ [M]$^+$: 539.2294. Found: 539.2298.

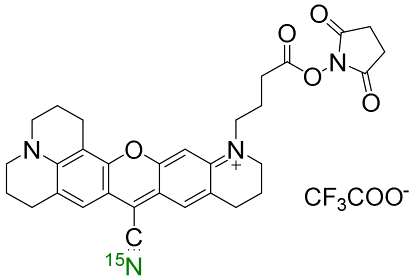

MARS2210-NHS (6b). ^{1}H NMR (400 MHz, MeOD) δ 7.55 (s, 1H), 7.50 (s, 1H), 7.05 (s, 2H), 3.74 (d, *J* = 7.3 Hz, 2H), 3.72 – 3.62 (m, 6H), 3.02 (t, *J* = 6.4 Hz, 2H), 2.99 – 2.92 (m, 4H), 2.87 (s, 4H), 2.84 (d, *J* = 6.8 Hz, 2H), 2.17 (p, *J* = 7.0 Hz, 2H), 2.07 (dt, *J* = 12.3, 5.7 Hz, 6H). ^{13}C NMR (101 MHz, Methanol-d_4) δ 170.4 , 168.6 , 156.0 , 154.0 , 152.8 , 151.3 , 127.6 , 127.0 , 126.1 , 124.7 ,

119.1 (d, J = 3.5 Hz), 115.3 , 112.5 , 112.2 (d, J = 16.9 Hz), 106.5 , 95.6 , 51.4 , 50.9 , 50.1 , 27.5 , 27.0 , 27.0 , 20.9 , 20.4 , 20.1 , 19.2 , 19.0. HRMS (ESI+) m/z Calcd. for $C_{31}H_{31}N_3{}^{15}NO_5{}^+$ $[M]^+$: 540.2265. Found: 540.2267.

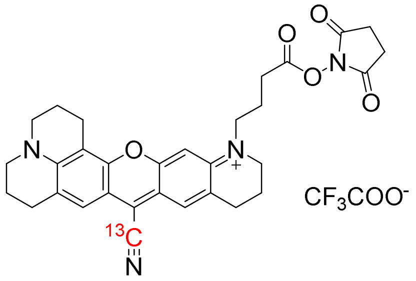

MARS2184-NHS (6c). ^{1}H NMR (400 MHz, Methanol-d_4) δ 7.53 (s, 1H), 7.48 (s, 1H), 7.04 (s, 1H), 3.74 (t, J = 7.3 Hz, 2H), 3.72 – 3.62 (m, 6H), 3.02 (t, J = 6.4 Hz, 2H), 3.00 – 2.92 (m, 5H), 2.87 (s, 4H), 2.84 (t, J = 6.8 Hz, 2H), 2.17 (p, J = 7.3 Hz, 2H), 2.13 – 2.00 (m, 6H). ^{13}C NMR (101 MHz, Methanol-d_4) δ 170.4 , 168.6 , 156.2 (d, J = 4.6 Hz), 154.0 , 152.8 , 151.4 (d, J = 4.8 Hz), 127.6 , 127.0 , 126.1 (d, J = 2.9 Hz), 124.7 (d, J = 3.3 Hz), 119.4 (d, J = 81.7 Hz), 115.4 (d, J = 1.8 Hz), 112.6 (d, J = 2.7 Hz), 112.3 , 106.5 , 95.7 , 51.3 , 50.8 , 50.1 , 27.5 , 27.0 , 20.9 , 20.4 , 20.1 , 19.2 , 19.0. HRMS (ESI+) m/z Calcd. for $C_{30}{}^{13}CH_{31}N_4O_5{}^+$ $[M]^+$: 540.2328. Found: 540.2329.

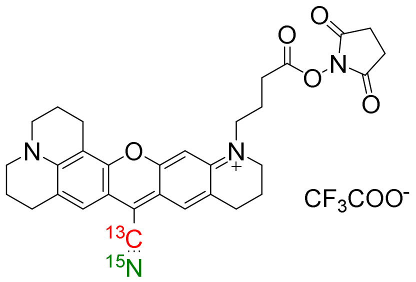

MARS2155-NHS (6d). ^{1}H NMR (400 MHz, MeOD) δ 7.56 (s, 1H), 7.51 (s, 1H), 7.07 (s, 1H), 3.76 (d, J = 7.3 Hz, 2H), 3.74 – 3.64 (m, 6H), 3.04 (t, J = 6.4 Hz, 2H), 3.02 – 2.94 (m, 4H), 2.89 (s, 4H), 2.86 (t, J = 6.9 Hz, 2H), 2.19 (p, J = 7.1 Hz, 2H), 2.15 – 2.03 (m, 6H). ^{13}C NMR (101 MHz, Methanol-d_4) δ 170.4 , 168.6 , 156.1 (d, J = 4.1 Hz), 154.0 , 152.8 , 151.3 (d, J = 4.9 Hz), 127.6 , 127.0 , 126.1 (d, J = 3.0 Hz), 124.7 (d, J = 3.4 Hz), 119.2 (dd, J = 81.7, 3.2 Hz), 115.3 (d, J = 1.6 Hz), 112.5 (d, J = 2.7 Hz), 112.2 (d, J = 17.3 Hz), 106.5 , 95.6 , 51.4 , 50.9 , 50.1 , 27.5 , 27.0 , 27.0 , 20.9 , 20.4 , 20.1 , 19.2 , 19.0. HRMS (ESI+) m/z Calcd. for $C_{30}{}^{13}CH_{31}N_3{}^{15}NO_5{}^+$ $[M]^+$: 541.2299. Found: 541.2301.

Synthesis of C-cored MARS NHS esters.

Supplementary Figure 13. Syntheses of C-cored MARS NHS esters. Counter ions for all NHS esters purified via HPLC are TFA anion.

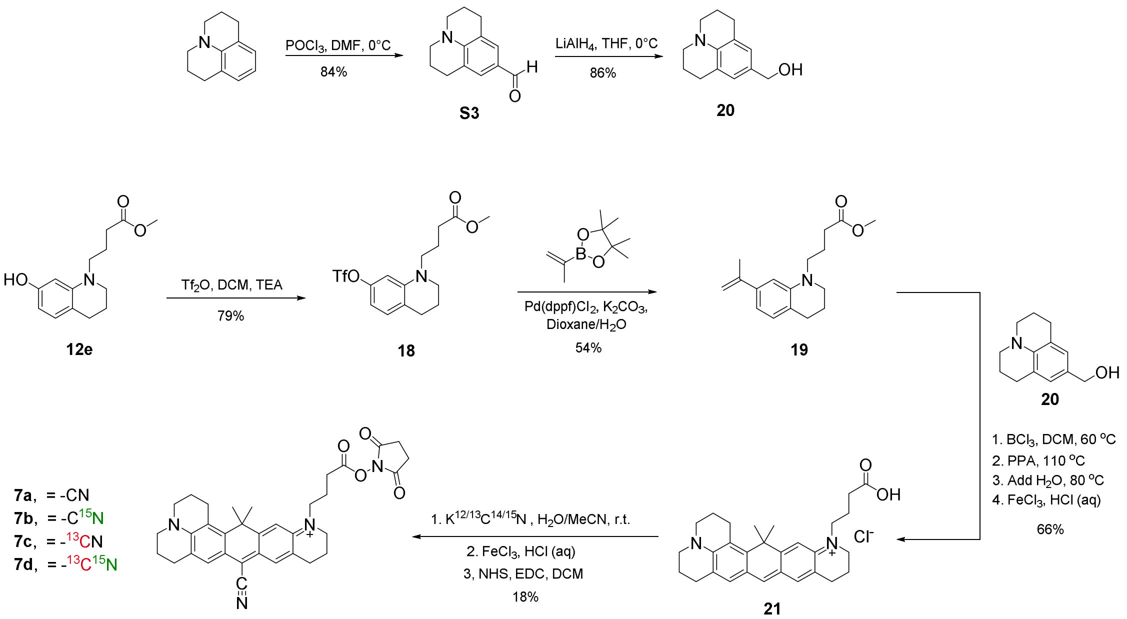

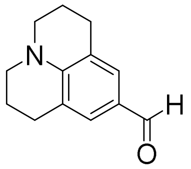

9-Formyl-2,3,6,7-tetrahydro-1H,5H-benzo[ij]-quinolizine (S3). Anhydrous DMF (10 mL) was placed into a round-bottom flask and cooled to 0 °C. $POCl_3$ (1.0 mL, 1.1 eq.) was then added dropwise while stirring. After 30 min, a solution of julolidine (1.73 g, 10.0 mmol) in 10 mL anhydrous DMF was added into flask. The reaction was then heated up to 90 °C for 5 h. After cooled to r.t., the reaction was quenched upon addition of 100 mL iced water and then the pH was adjusted to ~8 with solid $NaHCO_3$. The mixture was extracted with ethyl ether (50 mL ×3). The combined organic layers were dried over Na_2SO_4 and concentrated. The crude product was purified via silica gel column (eluent: DCM) to afford **S3** as yellowish oil (1.69 g, 84%). This compound has been characterized elsewhere showing a good match with our result.[2] ^{1}H NMR (400 MHz, $CDCl_3$) δ 9.62 (s, 1H), 7.31 (s, 2H), 3.35 – 3.27 (m, 4H), 2.79 (t, *J* = 6.3 Hz, 4H), 1.98 (dq, *J* = 7.0, 5.8 Hz, 4H).

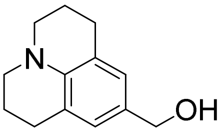

(2,3,6,7-tetrahydro-1H,5H-pyrido[3,2,1-ij]quinolin-9-yl)methanol (20). To a solution of **S3** (402 mg, 2.0 mmol) in 15 mL dry THF cooled at 0 °C was added $LiAlH_4$ (76 mg, 2.0 mmol, 1.0 eq) portionwise. The reaction was stirred for 1 h while allowed to warm up to room temperature. The reaction was quenched with 25 mL H_2O (caution when adding water!), filtered through a short pad of silica and washed with 30 mL DCM. The aqueous phase was further extracted with DCM (20 mL ×3). The combined organic layers were dried over Na_2SO_4 and evaporated to give **20** as yellow solid (348 mg, 86%). The product was sufficiently pure without further purification. Characterization

of of 10 was previously reported showing a good match with our result.[3] ^{1}H NMR (400 MHz, $CDCl_3$) δ 6.82 (s, 2H), 4.49 (s, 2H), 3.20 – 3.12 (m, 4H), 2.78 (t, *J* = 6.5 Hz, 4H), 2.05 – 1.94 (dq, *J* = 7.0, 5.8 Hz, 4H).

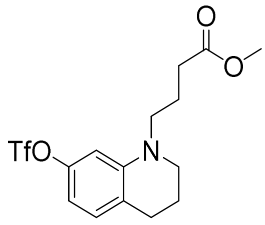

Methyl 4-(7-(((trifluoromethyl)sulfonyl)oxy)-3,4-dihydroquinolin-1(2H)-yl)butanoate (18). To a solution of **12e** (520 mg, 2.1 mmol) and triethylamine (1.16 mL, 8.4 mmol, 4 eq.) in anhydrous DCM (10 mL) was added trifluoromethanesulfonic anhydride (2.3 mL, 1 M solution in anhydrous DCM, 1.1 eq.) dropwise at 0 °C. After addition, the reaction was warmed up to r. t. and stirred for another 2 h before quenched with 1 N HCl (10 mL). The organic layer was separated and remaining aqueous layer was extracted with DCM (20 mL × 2). The combined organic layers were dried over Na_2SO_4 and evaporated in vacuo to give the crude product, which was further purified with silica gel flash chromatography (Hexane:EA = 10:1, v/v) to yield **18** as yellowish oil (606 mg, 79%).^{1}H NMR (400 MHz, $CDCl_3$) δ 6.91 (d, *J* = 8.4 Hz, 1H), 6.43 – 6.38 (m, 2H), 3.69 (s, 3H), 3.33 – 3.24 (m, 4H), 2.72 (t, *J* = 6.3 Hz, 2H), 2.37 (t, *J* = 7.1 Hz, 2H), 1.97 – 1.87 (m, 4H).

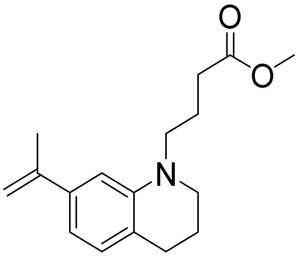

Methyl 4-(7-(prop-1-en-2-yl)-3,4-dihydroquinolin-1(2H)-yl)butanoate (19). Following a reported protocol[4], to a round-bottom flask was added **18** (376 mg, 0.98 mmol), Pd(dppf)$_2$Cl$_2$ (164 mg, 0.2 mmol, 20% eq.) and K_2CO_3 (276 mg, 2.0 mmol, 2 eq.). The flask was flushed with Ar followed by addition of 12 mL dioxane and 2 mL H_2O via syringe. Isopropenylboronic acid pinacol ester (282 μL, 1.50 mmol) was added into the flask and the reaction was stirred at 70 °C for 6 h before cooled to room temperature. The mixture was filtered through a short pad of silica, washed with DCM (100 mL) and the filtrate was evaporated to dry. The crude product was purified with silica gel flash chromatography (Hexane to Hexane: EA = 20:1, v/v) to give **19** as yellowish oil (145 mg, 54%). ^{1}H NMR (400 MHz, $CDCl_3$) δ 6.92 (d, *J* = 7.6 Hz, 1H), 6.75 – 6.66 (m, 2H), 5.31 (dd, *J* = 1.8, 0.9 Hz, 1H), 5.03 (p, *J* = 1.6 Hz, 1H), 3.70 (s, 3H), 3.38 – 3.32 (m, 2H), 3.31 – 3.27 (m, 2H), 2.76 (t, *J* = 6.4 Hz, 2H), 2.42 (t, *J* = 7.2 Hz, 2H), 2.15 (dd, *J* = 1.5, 0.8 Hz, 3H), 2.02 – 1.93 (m, 4H).

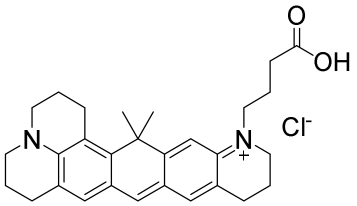

Pyronin intermediate 21. Following a previously reported protocol[4], **19** (145 mg, 0.53 mmol) and **10** (106 mg, 0.53 mmol) were dissolved in 10 mL dry DCM under Ar. To the mixture cooled to 0 °C was added BCl_3 (1 M in dry DCM) dropwise. The reaction was stirred overnight before a premixed solution of polyphosphoric acid (9.0 g) and H_3PO_4 (9.0 g, 85%) was added. The whole mixture was kept at 60 °C for 30 min until DCM thoroughly evaporated. The reaction was then heated to 110 °C and stirred vigorously for 4 h. After cooled to 80 °C, the reaction was treated with 20 mL H_2O, and kept stirring for additional 4 h until poured onto 30 g ice. The aqueous solution was neutralized carefully with 50% NaOH solution and extracted with DCM (30 mL ×3). To the combined organic phase were added 7.0 mL H_2O and 3.0 mL $FeCl_3$ solution (0.5 M in 1 N HCl) and the mixture was stirred vigorously for 2 h until the colorless intermediate was fully depleted. The organic layer was separated, dried over Na_2SO_4, and the solvent was removed in vacuo. The crude product was further purified with silica gel chromatography (DCM to DCM: MeOH = 20:1, v/v) to afford **21** as dark blue solid (168 mg, 66% overall). ^{1}H NMR (400 MHz, MeOD) δ ppm: 7.78 (s, 1 H), 7.27 (s, 1 H), 7.25 (s, 1 H), 7.23 (s, 1 H), 3.65-3.57 (m, 8 H), 3.17 (t, *J* = 5.8 Hz, 2 H), 2.80 (t, *J* = 5.8 Hz, 4 H), 2.48 (t, *J* = 6.4 Hz, 2 H), 2.04-2.00 (m, 8 H), 1.82 (s, 6 H). ^{13}C NMR (126 MHz, MeOD) δ ppm: 176.3, 160.0, 154.8, 154.5, 154.1, 151.1, 137.9, 135.5, 124.5, 124.5, 124.4, 122.7, 120.5, 111.6, 53.2, 52.5, 52.3, 51.5, 42.5, 31.1, 30.4, 28.3, 28.1, 27.8, 22.3, 22.2, 21.7, 21.6. HRMS (ESI+) m/z Calcd. for $C_{29}H_{35}N_2O_2$ $[M]^+$: 443.2699. Found: 443.2690

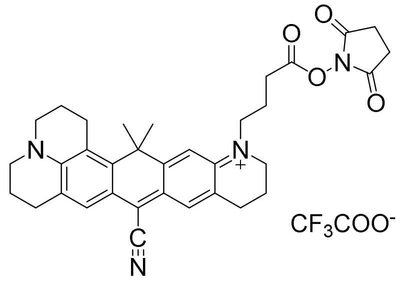

MARS2228-NHS (7a). Freshly prepared **21** (26 mg, 54 µmol) was dissolved in 6 mL MeCN and 2 mL H2O. 2 mL 0.1 M KCN aqueous solution was injected dropwise. The reaction was stirred at r.t. for 30 min and closely monitored with TLC until 11 was fully consumed and the solution turned to almost colorless. 2 mL FeCl3 (0.5 M solution in 1N HCl) was then added into the solution and the reaction was stirred for additional 1 h at r.t.. 20 mL brine was added and the aqueous phase was extracted with DCM (20 mL ×3) and combined organic layers were dried over Na_2SO_4 and concentrated. The crude product, N-hydroxylsuccinimide (NHS, 7.4 mg, 65 µmol, 1.2 eq.) and 1-ethyl-3-(3-dimethylaminopropyl)carbodiimide hydrochloride (EDC, 15.5 mg, 81 µmol, 1.5 eq.) were dissolved in 2 mL anhydrous DCM and the reaction was stirred at r.t. overnight before DCM was removed in vacuo. The crude product was purified by prep-HPLC (eluent: MeCN/H_2O, 20%→90%) to afford **7a** as dark green film (TFA salt, 6.8 mg, 18% over 3 steps). 1H NMR (400 MHz, MeOD) δ ppm: 7.62 (s, 1H), 7.49 (s, 1H), 7.06 (s, 1H), 3.79 (t, *J* = 7.8 Hz, 2H), 3.72-3.66 (m, 6H), 3.19 (t, *J* = 5.8 Hz, 2H), 2.88 (s, 4H), 2.88-2.85 (m, 6H), 2.17-2.12 (m, 2H), 2.07-2.05 (m, 6H), 1.82 (s, 6 H). ^{13}C NMR (126 MHz, DMSO-d6) δ ppm: 170.3, 168.9, 155.5, 153.4, 152.6, 147.2, 131.7, 129.9, 126.1, 125.6, 125.1, 124.0, 121.4, 117.5, 115.5, 111.4, 52.6, 51.8, 50.5, 50.4, 40.5, 30.1, 27.6, 26.7,

26.6, 26.2, 25.5, 21.7, 20.4, 19.8, 19.7. HRMS (ESI+) m/z Calcd. for $C_{34}H_{37}N_4O_4$ $[M]^+$: 565.2815. Found: 565.2812

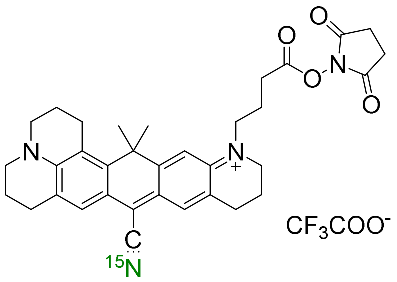

MARS2220-NHS ester (7b). ^{1}H NMR (400 MHz, MeOD) δ ppm: 7.63 (s, 1H), 7.50 (s, 1H), 7.07 (s, 1H), 3.79 (t, *J* = 7.8 Hz, 2H), 3.73-3.66 (m, 6H), 3.19 (m, 2H), 2.88 (s, 4H), 2.88-2.85 (m, 6H), 2.18-2.13 (m, 2H), 2.07-2.03 (m, 6H), 1.82 (s, 6H). HRMS (APCI+) m/z Calcd. for $C_{34}H_{37}N_3{}^{15}NO_4$ $[M]^+$: 566.2785. Found: 566.2791

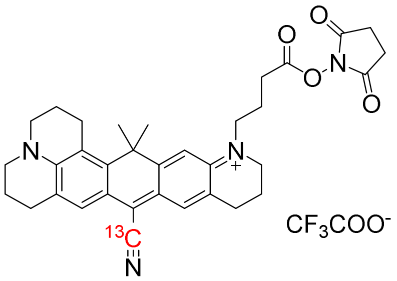

MARS2176-NHS ester (7c). ^{1}H NMR (400 MHz, MeOD) δ ppm: 7.63 (s, 1H), 7.51 (s, 1H), 7.07 (s, 1H), 3.79 (t, *J* = 8.0 Hz, 2H), 3.73-3.67 (m, 6H), 3.19 (t, *J* = 6.0 Hz, 2H), 2.88 (s, 4H), 2.88-2.85 (m, 6H), 2.18-2.15 (m, 2H), 2.09-2.03 (m, 6H), 1.82 (s, 6H). ^{13}C NMR (101 MHz, MeOD) δ 116.3. HRMS (ESI+) m/z Calcd. for $C_{33}{}^{13}CH_{37}N_4O_4$ $[M]^+$: 566.2848. Found: 566.2859

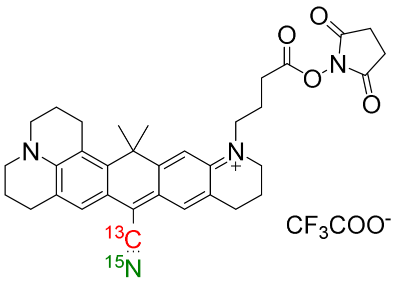

MARS2147-NHS ester (7d). ^{1}H NMR (400 MHz, MeOD) δ ppm: 7.63 (s, 1H), 7.51 (s, 1H), 7.07 (s, 1H), 3.79 (t, *J* = 7.8 Hz, 2H), 3.73-3.67 (m, 6H), 3.19 (t, *J* = 6.0 Hz, 2H), 2.88 (s, 4H), 2.88-2.85 (m, 6H), 2.18-2.12 (m, 2H), 2.09-2.03 (m, 6H), 1.82 (s, 6H). ^{13}C NMR (101 MHz, MeOD) δ 116.2 (d, *J* = 16.8 Hz). HRMS (ESI+) m/z Calcd. for $C_{33}{}^{13}CH_{37}N_3{}^{15}NO_4$ $[M]^+$: 567.2819. Found: 567.2825

Synthesis of Si-cored MARS NHS esters

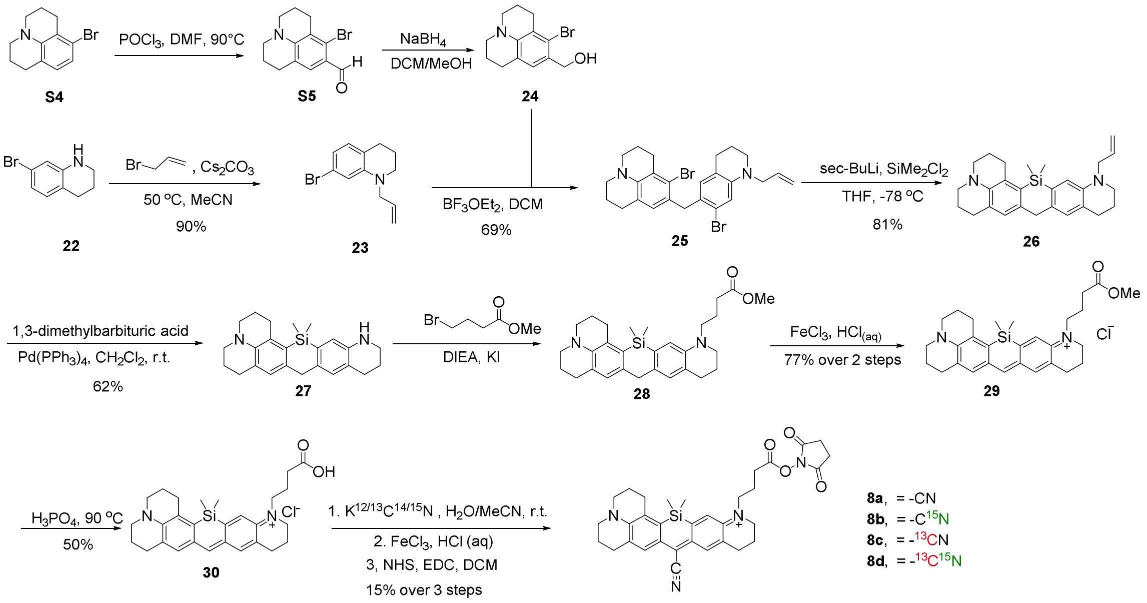

Supplementary Figure 14. Syntheses of Si-cored MARS NHS esters. Counter ions for all NHS esters purified via HPLC are TFA anion.

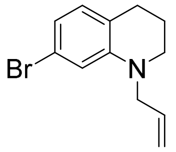

1-allyl-7-bromo-1,2,3,4-tetrahydroquinoline (23). 7-Bromo-1,2,3,4-Tetrahydroquinoline (1.0 g, 4.7 mmol) and cesium carbonate (7.6 g, 23.5 mmol, 5 eq.) was suspended in acetonitrile (100 mL). Allyl bromide (0.61mL, 7.05 mmol, 1.5 eq) was added in dropwise. The reaction was heated to 50 °C and stirred for 24 h before another portion of allyl bromide (0.61mL, 7.05 mmol) was added and stirred for another 24 h. The suspension was filtrated before the filtrate was concentrated and residue was purified by silica gel chromatography (EtOAc/Hexane= 5%) to obtain **23** as colorless oil (1.07 g, 90%). ^{1}H NMR (400 MHz, $CDCl_3$) δ 6.78 (dt, *J* = 7.8, 1.0 Hz, 1H), 6.69 – 6.61 (m, 2H), 5.89 – 5.75 (m, 1H), 5.24 – 5.13 (m, 2H), 3.84 (dt, *J* = 4.9, 1.8 Hz, 2H), 3.32 – 3.23 (m, 2H), 2.69 (t, *J* = 6.3 Hz, 2H), 1.99 – 1.87 (m, 2H). ^{13}C NMR (101 MHz, $CDCl_3$) δ 146.6, 132.8, 130.2, 121.3, 120.8, 118.3, 116.3, 113.5, 53.7, 49.0, 27.9, 22.1.

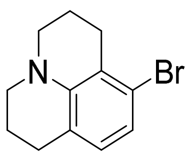

8-bromo-2,3,6,7-tetrahydro-1H,5H-pyrido[3,2,1-ij]quinoline (S4). S4 was synthesized based on a previously published protocol.[5]

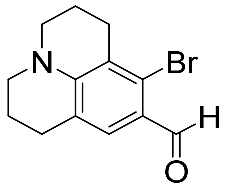

8-bromo-2,3,6,7-tetrahydro-1H,5H-pyrido[3,2,1-ij]quinoline-9-carbaldehyde (S5). Anhydrous DMF (2.6 mL) was charged in a flask under Argon and cool to 0 °C before $POCl_3$ was added dropwise. The reaction was stirred under 0 °C for 30 min and **S4** (506 mg, 2 mmol) in dry DMF (2.6 mL) was added. The reaction was then heated to 90 °C and stirred for 5 hours before poured to ice. The mixture was then added saturated $NaHCO_3$ solution to neutral and evaporated. The residue was re-dissolved in water, extract with methylene chloride, dried over Na_2SO_4 and evaporated. The residue was then purified by silica gel flash chromatography (EtOAc/Hexane= 0% -20%) to obtain **S5** (478 mg, 85%) as white solid. ^{1}H NMR (400 MHz, $CDCl_3$) δ 10.10 (s, 1H), 7.43 (d, *J* = 1.0 Hz, 1H), 3.33 – 3.23 (m, 4H), 2.84 (t, *J* = 6.5 Hz, 2H), 2.74 – 2.67 (m, 2H), 2.02 – 1.87 (m, 4H). ^{13}C NMR (101 MHz, $CDCl_3$) δ 191.1, 149.0, 129.7, 128.5, 121.6, 119.8, 119.4, 50.3, 49.9, 28.2, 27.5, 21.3, 21.1.

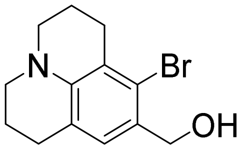

(8-bromo-2,3,6,7-tetrahydro-1H,5H-pyrido[3,2,1-ij]quinolin-9-yl)methanol (24). S5 (458 mg, 1.63 mmol) was dissolved in methylene chloride (4 mL) and methanol (4 mL) and cooled to 0 ℃ before sodium borohydride (296 mg, 2.4 mmol) was added in portion. The reaction was then warmed to room temperature and stirred for 30 minutes. Water was added to quench the reaction and the resulting mixture was extracted with ethyl acetate. The combined organic layers were then washed with water and brine, dried over Na_2SO_4 and evaporated. The residue was further purified by silica gel flash chromatography (Ethyl acetate/Hexane= 0% -20%) to obtain **24** (394.8 mg, 86%) as white solid.^{1}H NMR (400 MHz, $CDCl_3$) δ 6.86 (s, 1H), 4.61 (d, *J* = 5.5 Hz, 2H), 3.12 (dt, *J* = 16.4, 5.6 Hz, 4H), 2.80 (t, *J* = 6.7 Hz, 2H), 2.71 (t, *J* = 6.5 Hz, 2H), 2.01 – 1.92 (m, 4H). ^{13}C NMR (101 MHz, $CDCl_3$) δ 144.3, 128.3, 127.1, 124.3, 121.3, 120.7, 66.1, 50.1, 49.6, 29.0, 27.6, 22.1, 21.9.

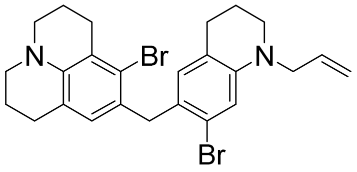

9-((1-allyl-7-bromo-1,2,3,4-tetrahydroquinolin-6-yl)methyl)-8-bromo-2,3,6,7-tetrahydro-1H, 5H-pyrido[3,2,1-ij]quinoline (25). 23 (646.3 mg, 3.18 mmol) and **24** (801.8 g, 3.18 mmol) was dissolved in dichloromethane (15 mL). BF_3•OEt_2 complex (628 μL, 5.1 mmol) was added to the solution at 0 °C. The reaction mixture was refluxed overnight. The saturated $NaHCO_3$ aqueous solution was added to it after cooling to room temperature. The aqueous phase was extracted with dichloromethane and the combined organic phase was washed with brine and dried over Na_2SO_4. After filtration, the filtrate was evaporated and the residue was purified by silica gel flash chromatography (EtOAc /Hexane =5%) to obtain **25** (1.13 g, 69 %) as colorless oil. ^{1}H NMR (400 MHz, $CDCl_3$) δ 6.74 (s, 1H), 6.60 (d, *J* = 4.6 Hz, 1H), 6.48 (s, 1H), 5.84 (ddt, *J* = 17.2, 10.2, 5.0 Hz, 1H), 5.25 – 5.13 (m, 2H), 3.93 (s, 2H), 3.83 (dt, *J* = 5.1, 1.7 Hz, 2H), 3.30 – 3.17 (m, 2H), 3.09 (dt,

J = 11.4, 5.6 Hz, 4H), 2.83 (t, *J* = 6.7 Hz, 2H), 2.63 (q, *J* = 7.0 Hz, 4H), 2.05 – 1.86 (m, 6H). ^{13}C NMR (101 MHz, $CDCl_3$) δ 144.8, 143.1, 133.2, 130.8, 128.6, 127.4, 126.5, 125.7, 123.0, 122.1, 121.4, 120.9, 116.4, 114.5, 54.0, 50.3, 49.7, 49.0, 40.9, 29.6, 27.8, 27.6, 22.4, 22.3, 22.1.

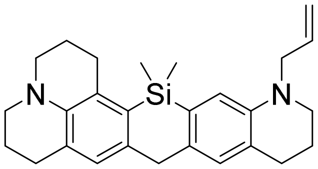

Reduced Si-pyronin precursor 26. 25 (1.13 g, 2.18 mmol) was dissolved in dry THF (60 mL) under Argon. The solution was cooled to -78 °C before sec-Butyl lithium hexane solution (1.4 M, 5.1 mL, 7.14 mmol) was added dropwise over 8 minutes. The reaction was left stirring under -78 °C for 20 minutes and then dichlorodimethylsilane (523.6 µL, 4.35 mmol) in dry THF (12mL) was slowly added. The reaction mixture was warmed up to room temperature, stirred for 3h and quenched by adding 1N HCl solution. Saturated $NaHCO_3$ solution was added to basify it, and the mixture was extracted with ethyl acetate. The organic phase was washed again with saturated $NaHCO_3$ solution, dried over Na_2SO_4 and evaporated. The residue was purified by silica gel flash chromatography (EtOAc/ Hexane = 2%, with 0.5% Et_3N) to obtain **26** (734.2 mg, 81 %) as colorless oil. ^1H NMR (400 MHz, $CDCl_3$) δ 6.87 (s, 1H), 6.79 (s, 1H), 6.76 (s, 1H), 5.89 (ddt, *J* = 17.3, 10.2, 5.2 Hz, 1H), 5.28 – 5.13 (m, 2H), 3.91 (s, 2H), 3.35 – 3.20 (m, 2H), 3.14 – 3.02 (m, 6H), 2.93 (t, *J* = 6.5 Hz, 2H), 2.88 – 2.71 (m, 4H), 2.23 – 1.82 (m, 8H), 0.47 (s, 6H). ^{13}C NMR (101 MHz, $CDCl_3$) δ 143.3, 141.1, 134.5, 134.2, 133.8, 133.5, 131.5, 128.2, 127.7, 127.5, 124.1, 123.6, 116.2, 115.9, 54.5, 50.8, 50.1, 49.6, 39.8, 29.6, 28.3, 28.1, 22.8, 22.6, 22.4, -0.2.

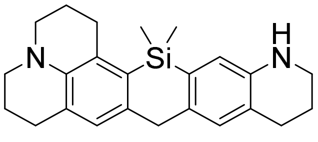

Reduced Si-pyronin precursor 27. 26 (722 mg, 1.74 mmol), 1,3-dimethylbarbituric acid (2.28 g, 14.6 mmol) and tetrakis(triphenylphosphine)palladium (386 mg, 0.33 mmol) was charged in a flask under argon. Deoxygenated methylene chloride (22 mL) was added and the reaction was stirred at room temperature overnight. Saturated $NaHCO_3$ solution was added and the reaction mixture was extracted with methylene chloride and the combined organic layers were washed with water and saturated $NaHCO_3$ before evaporation. The residue was purified by silica gel flash chromatography (Ethyl acetate/ Hexane = 5%- 15%, with 0.5% Et_3N) to obtain **27** (402.4 mg, 62%) as light blue oil. ^1H NMR (400 MHz, $CDCl_3$) δ 6.88 (s, 1H), 6.77 (s, 1H), 6.72 (s, 1H), 3.90 (s, 2H), 3.74 (br, 1H), 3.34 – 3.25 (m, 2H), 3.16 – 3.08 (m, 4H), 2.94 (t, *J* = 6.5 Hz, 2H), 2.81 – 2.72 (m, 4H), 2.22 – 1.78 (m, 6H), 0.47 (s, 6H). ^{13}C NMR (101 MHz, $CDCl_3$) δ 142.6, 141.1, 135.0, 134.5, 133.7, 131.5, 128.4, 127.7, 127.6, 123.6, 123.0, 119.1, 50.7, 50.1, 42.4, 40.1, 29.7, 28.1, 27.1, 22.8, 22.6, 22.3, -0.3.

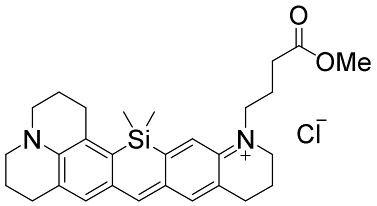

Si-pyronin 29. **27** (259.6 mg, 0.69 mmol) was dissolved in acetonitrile (11 mL) under argon. N,N-diisopropylethylamine (361 μL, 2.08 mmol), methyl 4-bromobutyrate (719 μL, 5.76 mmol) was added and the reaction was refluxed at 80 °C overnight. Water was added after cooling to room temperature. The mixture was extracted with DCM and the combined organic layer was washed with 2 N HCl for three time, dried over Na_2SO_4 and evaporated. $FeCl_3$ solution in HCl/H_2O (0.5 M in 1 N HCl, 13.6 mL, 6.8 mmol) was added to the residue. The reaction was stirred at room temperature overnight before extracting with methylene chloride. The combined organic layers were washed 2 N HCl, dried over Na_2SO_4 and evaporated. The residue was purified by silica gel flash chromatography (MeOH/CH_2Cl_2 = 3%-10%) to obtain **29** (273.6 mg, 77%) as dark blue solid. ^{1}H NMR (400 MHz, MeOD) δ 7.54 (s, 1H), 7.38 (s, 1H), 7.30 (s, 1H), 7.29 (s, 1H), 3.70 (s, 3H), 3.67 – 3.58 (m, 8H), 2.98 (dd, *J* = 7.1, 5.4 Hz, 2H), 2.78 (t, *J* = 6.3 Hz, 4H), 2.50 (t, *J* = 6.4 Hz, 2H), 2.11 – 1.96 (m, 8H), 0.59 (s, 6H). ^{13}C NMR (101 MHz, MeOD) δ 175.3, 159.0, 153.1, 152.6, 147.0, 142.6, 141.9, 140.2, 134.3, 129.1, 128.6, 126.0, 125.4, 120.4, 53.0, 52.4, 52.2, 52.2, 51.7, 31.1, 29.8, 28.3, 28.1, 22.6, 22.2, 21.9, 21.7, -1.2.

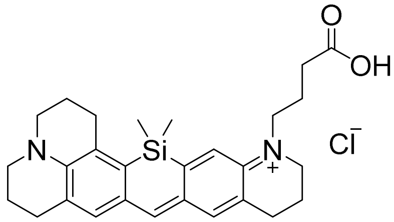

Si-pyronin 30. Freshly prepared **29** (273.6 mg, 0.54 mmol) was dissolved in phosphoric acid (20 mL, 85% wt. in water) and water (20 mL) and heated to 80°C. The reaction was stirred for 5h and cooled to room temperature before water was added to dilute the acidic mixture. The reaction was then extracted with methylene chloride. The combined organic layer was washed with brine, dried over Na_2SO_4 and evaporated. The residue was then purified by flash silica gel chromatography (MeOH: CH_2Cl_2 = 5%- 10%) to obtain **30** as blue solid (135.9 mg, 50%). ^{1}H NMR (400 MHz, MeOD) δ 7.54 (s, 1H), 7.40 (s, 1H), 7.30 (s, 1H), 7.28 (s, 1H), 3.73 – 3.56 (m, 8H), 3.02 – 2.94 (m, 2H), 2.78 (t, *J* = 6.2 Hz, 4H), 2.50 – 2.42 (m, 2H), 2.11 – 1.95 (m, 2H), 0.58 (s, 6H). ^{13}C NMR (101 MHz, MeOD) δ 177.4, 159.0, 153.2, 152.5, 147.1, 142.5, 141.9, 140.3, 134.1, 129.1, 128.6, 126.0, 125.3, 120.5, 53.0, 52.5, 52.3, 51.8, 30.7, 29.8, 28.3, 28.2, 23.4, 22.2, 21.9, 21.7, -1.2.

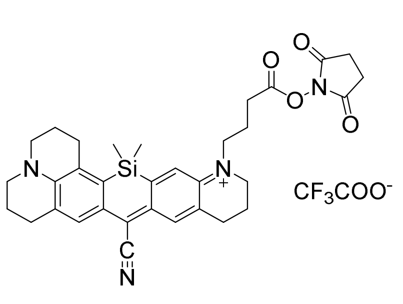

MARS2222-NHS ester (8a). 30 (6 mg, 0.012 mmol) was dissolved in acetonitrile (2 mL) and cooled to 0 °C before KCN solution in H_2O (0.1 M, 360 uL, 0.036 mmol) was added dropwise. The reaction was stirred under 0 °C for 20 minutes. $FeCl_3$ solution in HCl/H_2O (1 M in 1N HCl, 360 µL, 0.36 mmol) was added and the reaction was stirred at room temperature for 1 h. The reaction was extracted with methylene chloride and the combined organic layer was washed with brine, dried over Na_2SO_4 and evaporated. The residue was purified over a flash silica gel chromatography (MeOH: CH_2Cl_2=10%).

The obtained green solid together with N-hydroxylsuccinimide (3.6 mg, 0.031 mmol) and 1-ethyl-3-(3-dimethylaminopropyl)carbodiimide (8.4 mg, 0.44 mmol) was dissolved in dichloromethane anhydrous (2 mL) and stirred overnight. The reaction was then diluted with DCM, washed brine and dried over Na_2SO_4. The solvent was evaporated under vacuum and residue was purified through HPLC to obtain **8a** as green film (1.3 mg, 15.6%). ^{1}H NMR (400 MHz, MeOD) δ 7.80 (s, 1H), 7.73 (s, 1H), 7.26 (s, 1H), 3.87 – 3.65 (m, 8H), 3.06 – 2.95 (m, 2H), 2.92 – 2.80 (m, 10H), 2.21 – 1.95 (m, 8H), 0.58 (s, 6H). ^{13}C NMR (101 MHz, MeOD) δ 171.8, 170.4, 162.3, 153.1, 152.6, 145.1, 140.8, 138.3, 137.0, 136.4, 130.4, 130.2, 128.2, 127.8, 121.3, 118.0, 54.0, 53.2, 52.1, 51.8, 29.9, 28.7, 28.4, 28.2, 26.6, 23.0, 22.1, 21.8, 21.5, -1.0. HRMS (ESI+) m/z Calcd. for $C_{33}H_{37}N_4O_4Si^+$ $[M]^+$: 581.2584. Found: 581.2581.

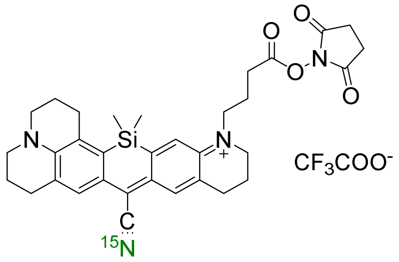

MARS2200-NHS ester (8b). 8b was obtained from **30** following same protocol as stated above for **8a** except using KC^{15}N. ^{1}H NMR (400 MHz, MeOD) δ 7.79 (s, 1H), 7.71 (s, 1H), 7.26 (s, 1H), 3.80 (t, *J* = 8.2 Hz, 2H), 3.71 (q, *J* = 6.2 Hz, 6H), 3.00 (t, *J* = 6.2 Hz, 2H), 2.91 – 2.80 (m, 10H), 2.24 – 1.95 (m, 8H), 0.57 (s, 6H). ^{13}C NMR (126 MHz, MeOD) δ 171.8, 170.5, 162.4, 153.1, 152.5, 145.0, 140.7, 138.3, 137.0, 136.3, 130.3 (d, *J*=3.0 Hz), 130.2, 128.2, 127.7, 121.3, 118.0 (d, *J*=16.4 Hz), 54.0, 53.2, 52.1, 51.8, 29.9, 28.7, 28.4, 28.2, 26.5, 23.0, 22.1, 21.8, 21.5, -1.0.

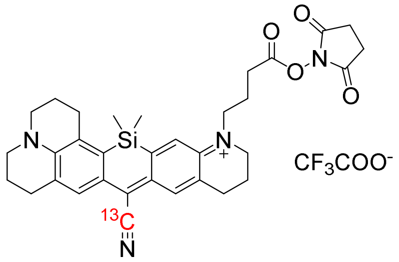

MARS2176-NHS ester (8c). ^{1}H NMR (500 MHz, MeOD) δ 7.81 (s, 1H), 7.73 (s, 1H), 7.26 (s, 1H), 3.80 (t, *J* = 8.2 Hz, 2H), 3.75 – 3.68 (m, 8H), 3.00 (t, *J* = 6.3 Hz, 2H), 2.90 – 2.83 (m, 10H), 2.18 – 1.99 (m, 8H), 0.58 (s, 6H). ^{13}C NMR (126 MHz, MeOD) δ 171.8, 170.5, 162.2, 153.0, 152.5, 144.8

(d, *J*= 5 Hz), 140.7, 138.3 (d, *J*= 5 Hz), 137.0, 136.4 (d, *J*= 4 Hz), 130.4, 130.2, 128.2, 127.8, 121.3, 118.0, 54.0, 53.2, 52.1, 51.8, 29.9, 28.7, 28.4, 28.2, 26.5, 23.0, 22.1, 21.8, 21.5, -1.0.

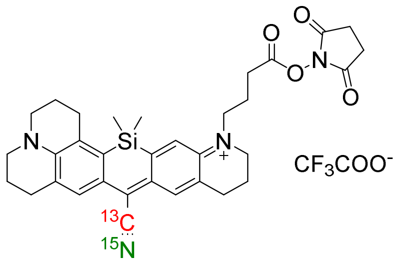

MARS2147-NHS ester (8d). ^{1}H NMR (500 MHz, MeOD) δ 7.79 (s, 1H), 7.72 (s, 1H), 7.26 (s, 1H), 3.80 (t, *J* = 8.2 Hz, 2H), 3.72 (p, *J* = 6.2 Hz, 6H), 3.00 (t, *J* = 6.3 Hz, 2H), 2.96 – 2.79 (m, 10H), 2.26 – 1.91 (m, 8H), 0.57 (s, 6H). ^{13}C NMR (126 MHz, MeOD) δ 171.8, 170.5, 162.3, 153.1, 152.5, 145.0 (d, *J*=5 Hz), 140.7 (d, *J*= 6 Hz), 138.3 (d, *J*= 4 Hz), 137.0, 136.3 (d, *J*= 3 Hz), 130.9, 130.2, 128.2, 127.8, 121.3, 118.0 (d, *J*= 18 Hz), 54.0, 53.2, 52.1, 51.8, 29.9, 28.7, 28.4, 28.2, 26.5, 23.0, 22.1, 21.8, 21.5, -1.0.

Synthesis of functionalized MARS probes for multiplexed imaging

Supplemental Figure 15. Syntheses of functionalized MARS probes for targeted labeling and multiplexed imaging.

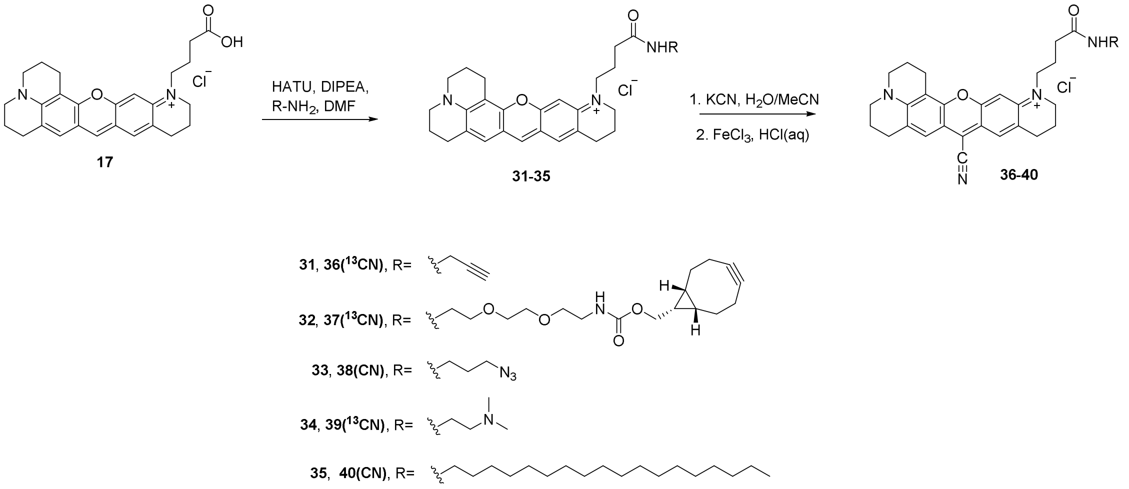

General methods for conjugated pyronins (31-35) and MARS probes (36-40) (*Method C*): A solution of **3i** (9.0 mg, 20 µmol) in 5 mL DMF was cooled to 0 °C followed by addition of 1-[Bis(dimethylamino)methylene]-1H-1,2,3-triazolo[4,5-b]pyridinium 3-oxide hexafluorophosphate (HATU, 45.6 mg, 120 µmol, 6 eq.), , and DIPEA (7.0 µL, 40 µmol, 2 eq.). The reaction was stirred for 10 min until the addition of corresponding amines (60 µmol, 3 eq.). The mixture was kept stirred overnight at room temperature and diluted with 20 mL saturated brine. The aqueous phase was extracted with DCM (20 mL ×3) and the combined organic layers were dried over Na_2SO_4 and concentrated. The residue was purified with silica gel chromatography (eluent: DCM to DCM: MeOH = 10:1, v/v). The obtained pyronins (**31-35**) were treated with ***method B*** to afford desired functionalized MARS probes (**36-40**) as dark blue solid.

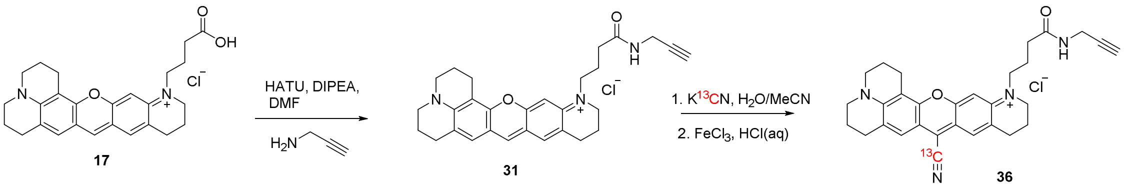

Pyronin intermediate for MARS2184-alkyne (31). ^{1}H NMR (500 MHz, MeOD) δ 8.25 (s, 1H), 7.43 (s, 1H), 7.35 (s, 1H), 6.93 (s, 1H), 4.01 (d, J = 2.5 Hz, 2H), 3.59 (dq, J = 10.7, 5.2 Hz, 8H), 3.05 (t, J = 6.3 Hz, 2H), 2.89 (t, J = 6.1 Hz, 4H), 2.60 (t, J = 2.5 Hz, 1H), 2.38 (t, J = 7.0 Hz, 2H), 2.11 – 2.00 (m, 8H). ^{13}C NMR (126 MHz, MeOD) δ 174.5, 158.5, 155.2, 154.0, 153.5, 144.3, 130.8, 129.5, 126.3, 125.9, 115.9, 114.9, 106.5, 96.3, 80.6, 72.2, 52.5, 52.1, 51.6, 51.2, 33.2, 29.5, 28.5, 28.4, 22.8, 22.0, 21.7, 20.7, 20.7.

MARS2184-alkyne (36). ^{1}H NMR (500 MHz, MeOD) δ 7.54 (s, 1H), 7.50 (s, 1H), 7.04 (s, 1H), 4.01 (d, J = 2.5 Hz, 2H), 3.72 – 3.62 (m, 8H), 3.06 (t, J = 6.4 Hz, 2H), 3.00 – 2.95 (m, 4H), 2.60 (t, J = 2.6 Hz, 1H), 2.39 (t, J = 6.9 Hz, 2H), 2.15 – 2.03 (m, 8H). ^{13}C NMR (126 MHz, MeOD) δ 174.4, 157.5 (d, J = 4.4 Hz), 155.5, 154.1, 152.8 (d, J = 4.6 Hz), 128.8, 128.5, 127.4 (d, J = 3.2 Hz), 126.1 (d, J = 3.2 Hz), 120.7 (d, J = 82.4 Hz), 116.5 (d, J = 2.0 Hz), 114.1 (d, J = 2.1 Hz), 113.7, 107.9, 97.2, 80.6, 72.3, 52.9, 52.7, 52.2, 51.6, 33.0, 29.5, 28.4, 28.4, 22.9, 21.9, 21.5, 20.7, 20.5. HRMS (ESI+) m/z Calcd. for $C_{29}{}^{13}CH_{31}N_4O_2^+$ [M]$^+$: 480.2480. Found: 480.2486.

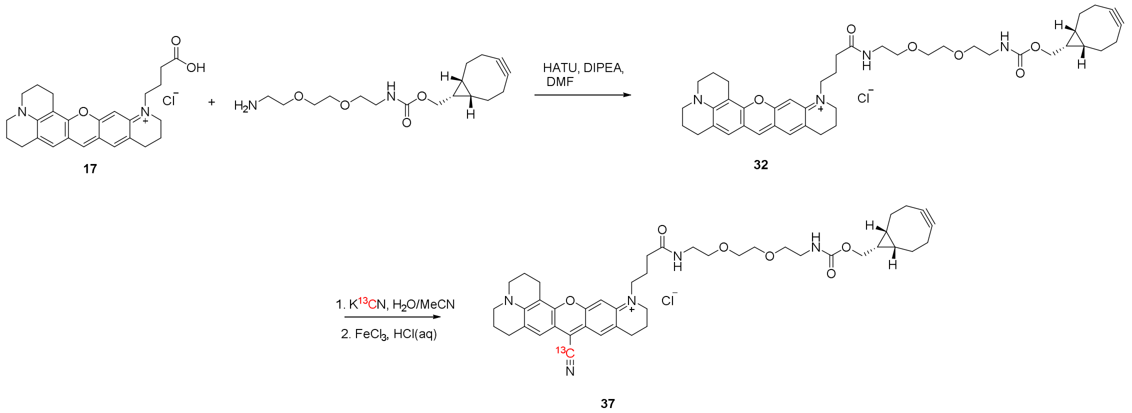

Pyronin intermediate for MARS2184-PEG2-Alkyne (32). ^{1}H NMR (500 MHz, CD_3CN) δ 8.14 (s, 1H), 7.38 (s, 1H), 7.30 (s, 1H), 6.94 (s, 1H), 6.64 (s, 1H), 5.63 (s, 1H), 4.07 (d, J = 8.0 Hz, 2H), 3.58 – 3.50 (m, 14H), 3.48 (t, J = 5.5 Hz, 2H), 3.37 (q, J = 5.5 Hz, 2H), 3.24 (q, J = 5.7 Hz, 2H), 2.97 (t, J = 6.4 Hz, 2H), 2.87 – 2.81 (m, 4H), 2.30 (t, J = 7.0 Hz, 2H), 2.27 – 2.10 (m, 6H), 2.04 (dt, J = 12.5, 6.7 Hz, 2H), 2.02 – 1.95 (m, 8H), 1.53 (q, J = 11.1, 9.8 Hz, 2H), 1.33 – 1.26 (m, 1H), 0.94 – 0.85 (m, 2H). ^{13}C NMR (126 MHz, CD_3CN) δ 173.4, 158.5, 158.2, 155.2, 153.9, 153.5, 144.2, 130.8, 129.5, 126.4, 125.9, 115.7, 114.7, 106.5, 100.1, 96.5, 71.3, 71.3, 71.0, 70.8, 63.5, 52.8, 52.2, 51.8, 51.3, 41.9, 40.3, 33.5, 30.2, 28.5, 28.4, 23.0, 22.2, 22.0, 21.7, 21.3, 20.8, 20.7, 19.1.

MARS2184-PEG2-Alkyne (37). ^{1}H NMR (500 MHz, MeOD) δ 7.55 (s, 1H), 7.50 (s, 1H), 7.03 (s, 1H), 4.08 (d, J = 8.1 Hz, 2H), 3.66 (q, J = 9.6, 7.6 Hz, 8H), 3.61 – 3.59 (m, 4H), 3.57 (t, J = 5.4 Hz, 2H), 3.50 (t, J = 5.6 Hz, 2H), 3.41 (t, J = 5.4 Hz, 2H), 3.24 (t, J = 5.6 Hz, 2H), 3.04 (t, J = 6.4 Hz, 2H), 2.96 (q, J = 6.0 Hz, 4H), 2.38 (t, J = 7.0 Hz, 2H), 2.26 – 2.15 (m, 4H), 2.14 – 2.01 (m, 10H), 1.59 – 1.48 (m, 2H), 1.37 – 1.30 (m, 1H), 0.94 – 0.82 (m, 2H). ^{13}C NMR (126 MHz, MeOD) δ 174.9, 159.2, 157.6 (d, J = 4.8 Hz), 155.5, 154.1, 152.8 (d, J = 4.5 Hz), 128.8, 128.5, 127.5 (d, J = 3.1 Hz), 126.2 (d, J = 3.2 Hz), 120.8 (d, J = 81.9 Hz), 116.6 (d, J = 2.1 Hz), 114.2 (d, J = 2.0 Hz), 113.7, 107.9, 99.5, 97.2, 71.3, 71.3, 71.0, 70.5, 63.7, 53.0, 52.7, 52.2, 51.6, 41.6, 40.4, 33.3, 30.1, 28.4, 28.4, 23.1, 22.0, 21.9, 21.5, 21.4, 20.7, 20.5, 18.9. HRMS (ESI+) m/z Calcd. for $C_{43}{}^{13}CH_{54}N_5O_6^+$ [M]$^+$: 749.4108. Found: 749.4110.

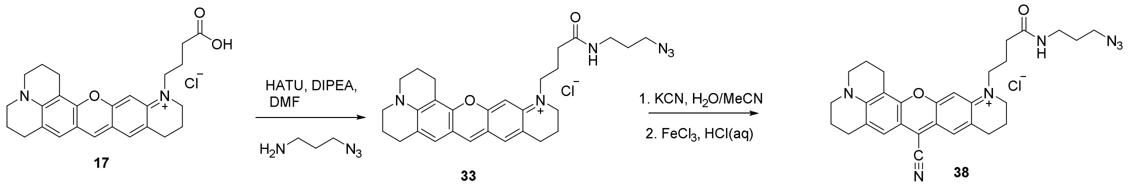

Pyronin intermediate for MARS2238-Azide (33). ^{1}H NMR (500 MHz, MeOD) δ 8.24 (s, 1H), 7.43 (s, 1H), 7.34 (s, 1H), 6.93 (s, 1H), 3.64 – 3.54 (m, 8H), 3.37 (t, *J* = 6.7 Hz, 2H), 3.30 (t, *J* = 6.9 Hz, 2H), 3.04 (t, *J* = 6.3 Hz, 2H), 2.89 (s, 4H), 2.37 (t, *J* = 7.0 Hz, 2H), 2.12 – 2.00 (m, 8H), 1.78 (p, *J* = 6.8 Hz, 2H). ^{13}C NMR (126 MHz, MeOD) δ 175.0, 158.5, 155.2, 153.9, 153.5, 144.3, 130.8, 129.5, 126.3, 125.9, 115.9, 114.8, 106.5, 96.3, 52.5, 52.1, 51.6, 51.1, 50.1, 37.8, 33.4, 29.7, 28.5, 28.4, 23.0, 22.0, 21.7, 20.7, 20.7.

MARS2238-Azide (38). ^{1}H NMR (400 MHz, MeOD) δ 7.54 (s, 1H), 7.50 (s, 1H), 7.04 (s, 1H), 3.73 – 3.62 (m, 8H), 3.37 (t, *J* = 6.7 Hz, 2H), 3.30 (d, *J* = 5.3 Hz, 2H), 3.05 (t, *J* = 7.4 Hz, 2H), 2.97 (s, 4H), 2.38 (t, *J* = 7.0 Hz, 2H), 2.08 (s, 8H), 1.78 (p, *J* = 7.6 Hz, 2H). ^{13}C NMR (101 MHz, MeOD) δ 174.9, 157.5, 155.6, 154.1, 152.8, 128.8, 128.5, 127.4, 126.1, 120.7, 116.5, 114.1, 113.7, 107.9, 97.2, 53.0, 52.7, 52.2, 51.5, 50.1, 37.8, 33.2, 29.7, 28.4, 28.4, 23.0, 21.9, 21.5, 20.7, 20.5. HRMS (ESI+) m/z Calcd. for $C_{30}H_{34}N_7O_2^+$ [M]$^+$: 524.2774. Found: 524.2772.

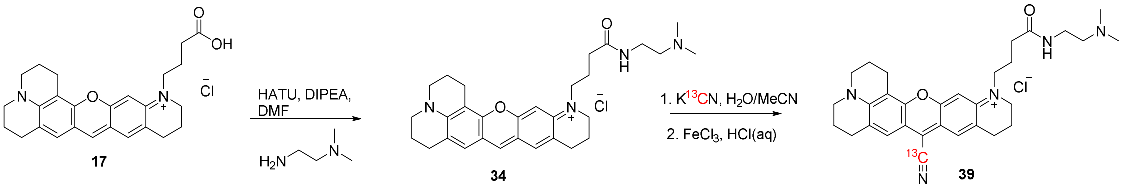

Pyronin intermediate for MARS2184-Lyso (34). ^{1}H NMR (400 MHz, MeOD) δ 8.29 (s, 1H), 7.46 (s, 1H), 7.38 (s, 1H), 6.99 (s, 1H), 3.62 (ddd, *J* = 18.7, 10.5, 5.0 Hz, 10H), 3.30 (d, *J* = 5.8 Hz, 2H), 3.06 (t, *J* = 6.4 Hz, 2H), 2.98 (s, 6H), 2.94 – 2.88 (m, 4H), 2.44 (t, *J* = 7.3 Hz, 2H), 2.14 – 2.00 (m, 8H). ^{13}C NMR (101 MHz, MeOD) δ 176.2, 158.5, 155.2, 153.9, 153.5, 144.3, 130.8, 129.5, 126.3, 125.9, 115.9, 114.8, 106.5, 96.3, 58.6, 52.6, 52.1, 51.6, 51.2, 43.9, 38.9, 35.8, 33.4, 28.5, 28.4, 22.7, 22.0, 21.7, 20.7, 20.7.

MARS2184-Lyso (39). ^{1}H NMR (400 MHz, MeOD) δ 7.57 (s, 1H), 7.52 (s, 1H), 7.07 (s, 1H), 3.74 – 3.64 (m, 8H), 3.61 (t, *J* = 5.6 Hz, 2H), 3.29 (d, *J* = 5.7 Hz, 2H), 3.04 (t, *J* = 6.0 Hz, 2H), 3.00 – 2.94 (m, 10H), 2.44 (t, *J* = 7.1 Hz, 2H), 2.16 – 2.02 (m, 8H). ^{13}C NMR (101 MHz, MeOD) δ 176.0, 157.6 (d, *J* = 4.5 Hz), 155.5, 154.1, 152.8, 128.8, 128.4, 127.5 (d, *J* = 3.0 Hz), 126.2 (d, *J* = 3.4 Hz), 120.8 (d, *J* = 81.9 Hz), 116.6 (d, *J* = 2.4 Hz), 114.1 (d, *J* = 2.3 Hz), 113.7, 107.8, 97.2, 58.5, 53.1, 52.7, 52.2, 51.6, 43.8, 35.7, 33.2, 28.4, 28.4, 22.8, 21.9, 21.5, 20.7, 20.4. HRMS (ESI+) m/z Calcd. for $C_{30}{}^{13}CH_{38}N_5O_2^+$ [M]$^+$: 513.3059. Found: 513.3063.

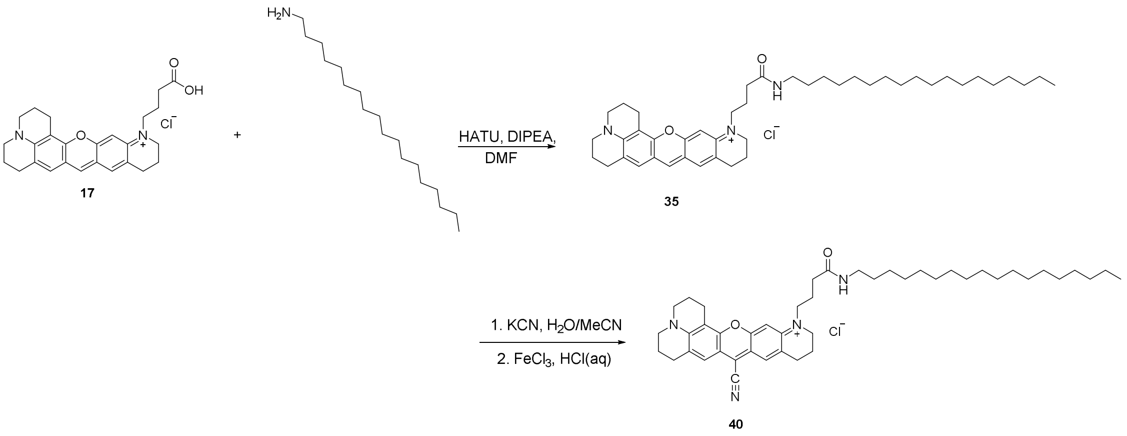

Pyronin intermediate for MARS2238-C18 (35). ^{1}H NMR (500 MHz, MeOD) δ 8.29 (s, 1H), 7.45 (s, 1H), 7.38 (s, 1H), 6.95 (s, 1H), 3.65 – 3.56 (m, 8H), 3.22 (t, *J* = 7.0 Hz, 3H), 3.07 (t, *J* = 6.3 Hz, 2H), 2.94 – 2.88 (m, 4H), 2.36 (t, *J* = 6.9 Hz, 2H), 2.13 – 2.00 (m, 8H), 1.51 (p, *J* = 7.1 Hz, 2H), 1.39 – 1.18 (m, 30H), 0.91 (t, *J* = 7.0 Hz, 3H). ^{13}C NMR (126 MHz, MeOD) δ 174.8, 158.6, 155.3, 154.0, 153.5, 144.4, 130.9, 129.6, 126.4, 126.0, 115.9, 114.9, 106.5, 96.3, 52.5, 52.1, 51.7, 51.1, 40.4, 33.5, 33.1, 30.8, 30.7, 30.7, 30.7, 30.5, 30.4, 28.5, 28.5, 28.0, 23.7, 23.0, 22.0, 21.7, 20.8, 20.7, 14.4.

MARS2238-C18 (40). ^{1}H NMR (500 MHz, MeOD) δ 7.56 (s, 1H), 7.51 (s, 1H), 7.04 (s, 1H), 3.73 – 3.63 (m, 8H), 3.22 (t, *J* = 7.0 Hz, 2H), 3.06 (t, *J* = 6.4 Hz, 2H), 3.00 – 2.95 (m, 4H), 2.37 (t, *J* = 6.9 Hz, 2H), 2.13 – 2.02 (m, 8H), 1.51 (p, *J* = 7.2 Hz, 2H), 1.37 – 1.21 (m, 30H), 0.92 (t, *J* = 7.0 Hz, 3H). ^{13}C NMR (126 MHz, MeOD) δ 174.7, 157.6, 155.6, 154.1, 152.8, 128.8, 128.5, 127.4, 126.1, 120.7, 116.5, 114.1, 113.7, 107.9, 97.2, 53.0, 52.7, 52.2, 51.5, 40.4, 33.3, 33.1, 30.8, 30.7, 30.7, 30.5, 30.4, 28.4, 28.4, 28.0, 23.7, 23.1, 21.9, 21.5, 20.7, 20.5, 14.4. HRMS (ESI+) m/z Calcd. for $C_{45}H_{65}N_4O_2^+$ [M]$^+$: 693.5107. Found: 693.5107.

References

1. Anzalone, A. V.; Wang, T. Y.; Chen, Z.; Cornish, V. W., A common diaryl ether intermediate for the gram-scale synthesis of oxazine and xanthene fluorophores. *Angew. Chem. Int. Ed. Engl.* **2013,** *52* (2), 650-4.
2. Wang, H.; Lu, Z.; Lord, S. J.; Willets, K. A.; Bertke, J. A.; Bunge, S. D.; Moerner, W. E.; Twieg, R. J., The influence of tetrahydroquinoline rings in dicyanomethylenedihydrofuran (DCDHF) single-molecule fluorophores. *Tetrahedron* **2007,** *63* (1), 103-114.
3. Bachman, J. L.; Pavlich, C. I.; Boley, A. J.; Marcotte, E. M.; Anslyn, E. V., Synthesis of Carboxy ATTO 647N Using Redox Cycling for Xanthone Access. *Org. Lett.* **2020,** *22* (2), 381-385.
4. Wei, L.; Chen, Z.; Shi, L.; Long, R.; Anzalone, A. V.; Zhang, L.; Hu, F.; Yuste, R.; Cornish, V. W.; Min, W., Super-multiplex vibrational imaging. *Nature* **2017.**
5. Roth, A.; Li, H.; Anorma, C.; Chan, J., A Reaction-Based Fluorescent Probe for Imaging of Formaldehyde in Living Cells. *J. Am. Chem. Soc.* **2015,** *137* (34), 10890-3.
