# Supplementary material for: 9-Cyanopyronin probe palette for super-multiplexed vibrational imaging
Source: Nat Commun. 2021 Jul 26;12:4518. doi: 10.1038/s41467-021-24855-6 (PMC8313527; doi:10.1038/s41467-021-24855-6)
Supplement: Supplementary file 3 — Reporting Summary [file 41467_2021_24855_MOESM3_ESM.pdf]

nature research

Corresponding author(s): Wei Min

Last updated by author(s): Jun 11, 2021

Reporting Summary

Nature Research wishes to improve the reproducibility of the work that we publish. This form provides structure for consistency and transparency in reporting. For further information on Nature Research policies, see our Editorial Policies and the Editorial Policy Checklist.

Statistics

For all statistical analyses, confirm that the following items are present in the figure legend, table legend, main text, or Methods section.

n/a	Confirmed	
[ ]	[x]	The exact sample size (*n*) for each experimental group/condition, given as a discrete number and unit of measurement
[ ]	[x]	A statement on whether measurements were taken from distinct samples or whether the same sample was measured repeatedly
[x]	[ ]	The statistical test(s) used AND whether they are one- or two-sided<br>*Only common tests should be described solely by name; describe more complex techniques in the Methods section.*
[ ]	[x]	A description of all covariates tested
[ ]	[x]	A description of any assumptions or corrections, such as tests of normality and adjustment for multiple comparisons
[x]	[ ]	A full description of the statistical parameters including central tendency (e.g. means) or other basic estimates (e.g. regression coefficient) AND variation (e.g. standard deviation) or associated estimates of uncertainty (e.g. confidence intervals)
[x]	[ ]	For null hypothesis testing, the test statistic (e.g. *F*, *t*, *r*) with confidence intervals, effect sizes, degrees of freedom and *P* value noted<br>*Give P values as exact values whenever suitable.*
[x]	[ ]	For Bayesian analysis, information on the choice of priors and Markov chain Monte Carlo settings
[x]	[ ]	For hierarchical and complex designs, identification of the appropriate level for tests and full reporting of outcomes
[x]	[ ]	Estimates of effect sizes (e.g. Cohen's *d*, Pearson's *r*), indicating how they were calculated

Our web collection on statistics for biologists contains articles on many of the points above.

Software and code

Policy information about availability of computer code

Data collection	All SRS spectral and imaging data were collected with commercial Olympus confocal microscopy software package FV3000.
Data analysis	All spectral data was analyzed with OriginPro (2016) software. Imaging data was analyzed and processed with ImageJ (v2.1.0) or Matlab (R2017b)

For manuscripts utilizing custom algorithms or software that are central to the research but not yet described in published literature, software must be made available to editors and reviewers. We strongly encourage code deposition in a community repository (e.g. GitHub). See the Nature Research guidelines for submitting code & software for further information.

Data

Policy information about availability of data

All manuscripts must include a data availability statement. This statement should provide the following information, where applicable:
- Accession codes, unique identifiers, or web links for publicly available datasets
- A list of figures that have associated raw data
- A description of any restrictions on data availability

The data that support the findings of the study are all provided with this paper in supplementary information or source data files.

Field-specific reporting

Life sciences study design

All studies must disclose on these points even when the disclosure is negative.

Sample size	No sample size calculation was performed in this study.<br>The imaging experiments in this study were not focused on any quantitative analysis. For all SRS imaging practices in Figure 3 and 4, all cells were grown on round coverslips with total cell number >50k, and at least 3 random fields of view (FoVs) containing at least 5 cells were selected to ensure the sampling is random and the sample sizes are sufficient. All images showed similar labeling patterns confirming the consistency among cells/tissue areas.
Data exclusions	No data was excluded.
Replication	The number of replicates for each individual experiment is indicated in the manuscripts. Cell labeling experiments were replicated every month, with a total replication number of at least 3. All replications were successful.
Randomization	We do not have statistical model presented and the imaging study does not focus on any quantitative analysis. The samples were not allocated into different experimental groups. Hence, randomization was not needed or performed.
Blinding	The study does not involve any comparisons between experimental groups, so the samples were not allocated into different experimental groups. Therefore, no blinding was needed.

Reporting for specific materials, systems and methods

We require information from authors about some types of materials, experimental systems and methods used in many studies. Here, indicate whether each material, system or method listed is relevant to your study. If you are not sure if a list item applies to your research, read the appropriate section before selecting a response.

Materials & experimental systems

n/a	Involved in the study	
☐	☒	Antibodies
☐	☒	Eukaryotic cell lines
☒	☐	Palaeontology and archaeology
☒	☐	Animals and other organisms
☒	☐	Human research participants
☒	☐	Clinical data
☒	☐	Dual use research of concern

Methods

n/a	Involved in the study	
☒	☐	ChIP-seq
☒	☐	Flow cytometry
☒	☐	MRI-based neuroimaging

Antibodies

Antibodies used	Primary Antibodies:<br>1)Anti-alpha Tubulin Monoclonal Antibody (mouse, Invitrogen, #A11126)<br>2)Anti-Fibrillarin Antibody [38F3] (mouse, Abcam, #ab4566)<br>3)Recombinant Alexa Fluor® 568 Anti-NeuN antibody [EPR12763] (Rabbit, Abcam, #ab207282)<br>4)Monoclonal Anti-Calbindin-D-28K antibody (mouse, Sigma-Aldrich, #c9848)<br>5)Anti-Vimentin antibody (chicken, Abcam, #ab24525)<br>6)Anti-Myelin Basic Protein antibody [12] (rat, Abcam, #ab7349)<br>7)Anti-GFAP antibody (goat, Abcam, #ab53554)<br>8)Anti-GABA B Receptor R2 Antibody (guinea pig, Sigma-Aldrich, #AB2255)<br>9)Fibrillarin Polyclonal Antibody (rabbit, Invitrogen, #PA5-29801)<br><br>Secondary Antibodies:<br>1)Donkey anti-Mouse IgG (H+L) Secondary Antibody (Invitrogen, #A16013)<br>2)Donkey anti-Rat IgG (H+L) Highly Cross-Adsorbed Secondary Antibody (Invitrogen, #A18747)<br>3)Donkey anti-Rabbit IgG (H+L) Cross-Adsorbed Secondary Antibody (Invitrogen, #A31238)<br>4)Donkey anti-Chicken IgY (H+L) Secondary Antibody (Invitrogen, #SA1-72002)<br>5)Donkey anti-Goat IgG (H+L) Cross-Adsorbed Secondary Antibody (Invitrogen, #A16007)<br>6)Anti-Guinea Pig IgG (H+L), highly cross adsorbed antibody produced in donkey (Sigma-Aldrich, #SAB3700384)<br>7)Goat anti-Mouse IgG (H+L) Secondary Antibody (Invitrogen, #31160)<br>8)Goat anti-Rabbit IgG (H+L) Cross-Adsorbed Secondary Antibody (Invitrogen, #31212)
Validation	1)The anti-bovine alpha-tubulin antibody was certified by Invitrogen to be used with secondary detection techniques to visualize microtubules in fixed cells and in fixed or frozen tissue sections of various species.<br>2)The Anti-Fibrillarin Antibody was authenticated by Abcam that it is suitable for immunolabeling of mammalian cells.

3)The NeuN-Alexa 568 antibody was authenticated by Abcam to be capable of labeling NeuN, a neuronal marker.
4)The Anti-Calbindin antibody was authenticated by Sigma-Aldrich to be capable of reacting with mouse calbindin.
5)The Anti-Vimentin antibody was authenticated by Abcam to react with mouse vimentin, a cytoskeleton marker.
6)The Anti-Myelin Basic Protein antibody was authenticated by Abcam to react with mouse MBP.
7)The Anti-GFAP antibody was authenticated by Abcam to react with mouse GFAP.
8)The Anti-GABA B Receptor R2 Antibody was authenticated by Sigma-Aldrich to react with mouse GABAR2 (predicted by homology).
9) The Anti-Fibrillarin antibody was authenticated by Invitrogen to bind with Fibrillarin.

Eukaryotic cell lines

Policy information about cell lines

Cell line source(s)	HeLa cells purchased from ATCC (CCL-2).
Authentication	Cells were authenticated by ATCC. The identification of short tandem repeat (STR) markers was performed for authentication.
Mycoplasma contamination	No mycoplasma was detected.
Commonly misidentified lines (See ICLAC register)	No commonly misidentified cell lines were used in the study.
